# The prognostic and therapeutic significance of polyunsaturated fatty acid‐derived oxylipins in ST‐segment elevation myocardial infarction

**DOI:** 10.1002/imt2.266

**Published:** 2025-01-09

**Authors:** Zhiyong Du, Yingyuan Lu, Ying Ma, Yunxiao Yang, Wei Luo, Sheng Liu, Ming Zhang, Yong Wang, Lei Li, Chun Li, Wei Wang, Hai Gao

**Affiliations:** ^1^ Key Laboratory of Remodeling‐Related Cardiovascular Diseases, Ministry of Education, National Clinical Research Center for Cardiovascular Diseases, Beijing Institute of Heart Lung and Blood Vessel Disease, Beijing Anzhen Hospital Capital Medical University Beijing China; ^2^ School of Pharmaceutical Sciences, State Key Laboratory of Natural and Biomimetic Drugs, Peking University Peking University Beijing China; ^3^ The State Key Laboratory for Quality Ensurance and Sustainable Use of Dao‐di Herbs, National Resource Center for Chinese Materia Medica, China, Academy of Chinese Medical Sciences Beijing China; ^4^ Department of Cardiology, Beijing Anzhen Hospital Capital Medical University Beijing China; ^5^ Dongzhimen Hospital of Beijing University of Chinese Medicine Beijing China; ^6^ Department of Cardiology Peking University Third Hospital Beijing China; ^7^ Institute of Traditional Chinese Medicine Beijing University of Chinese Medicine Beijing China; ^8^ Chinese Medicine Guangdong Laboratory Guangdong Hengqin China; ^9^ State Key Laboratory of Traditional Chinese Medicine Syndrome Guangzhou University of Chinese Medicine Guangzhou China

**Keywords:** functional metabolomics, inflammation, oxylipins, prognostic markers, ST‐segment elevation myocardial infarction

## Abstract

Polyunsaturated fatty acid‐derived oxylipins regulate systemic inflammation and exert cardiovascular effects, yet their role in ST‐segment elevation myocardial infarction (STEMI) remains unclear. Herein, we used targeted metabolomics and machine learning algorithms to develop an oxylipin‐based risk model to accurately predict recurrent major adverse cardiovascular events (MACE) after STEMI in two independent prospective cohorts with 2 years of follow‐up. The in vivo effects of significant oxylipin predictors were explored via a murine myocardial ischemia‒reperfusion model and functional metabolomics. Among the 130 plasma oxylipins detected in discovery cohort (*n* = 645), patients with and without recurrent MACE exhibited significant differences in a variety of oxylipin subclasses. We constructed an oxylipin‐based prediction model that showed powerful performance in predicting recurrent MACE in the discovery cohort (predictive accuracy: 91.5%). The predictive value of the oxylipin marker panel was confirmed in an independent external validation cohort (predictive accuracy: 89.9%; *n* = 401). Furthermore, we found that the anti‐inflammatory/pro‐resolving oxylipin (ARO) predictor panel showed better prognostic performance than the pro‐inflammatory oxylipin predictor panel in both cohorts. Compared with the treatment of pro‐inflammatory oxylipin predictor panel, combined treatment of six ARO predictors, including 14,15 epoxy‐eicosatrienoic acid, 14(15)‐epoxy‐eicosatetraenoic acid, 12,13‐epoxy‐octadecenoic acid, lipoxin A4, resolving D1, and 6 keto‐prostaglandin F1 showed significant cardiac activities and synergistic metabolic actions in myocardial infarction‒reperfusion model mice. We also mechanistically identified an important role of ARO predictors in restraining ceramide/lysophosphatidylcholine synthesis and inhibiting inflammatory responses. Overall, the present study depicted the landscape of oxylipin profiles in the largest panel of STEMI patients worldwide. Our results also highlight the great potential of bioactive oxylipins in prognostic prediction and therapeutics after STEMI.

## Highlights

- Polyunsaturated fatty acid‐derived oxylipins regulate systemic inflammation and exert cardiovascular effects, however, their role in ST‐segment elevation myocardial infarction (STEMI) remains unclear.
- We constructed an oxylipin‐based prediction model that showed powerful performance in predicting recurrent major adverse cardiovascular events in two independent cohorts of 1046 STEMI patients.
- We also demonstrated that six anti‐inflammatory/pro‐resolving oxylipin combinations had synergistic and cardioprotective effects against myocardial ischemia‒reperfusion injury.

## Graphical abstract

In this study, we comprehensively profiled plasma oxylipin alterations in patients with ST‐segment elevation myocardial infarction (STEMI) with and without recurrent major adverse cardiovascular events (MACE) in a prospective cohort (*n* = 645) with 2 years of follow‐up. Using a multivariate random forest algorithm, we identified a panel of 14 oxylipin markers that showed powerful performance in predicting recurrent MACE. The predictive performance of the oxylipin marker panel was confirmed in an independent external validation cohort (*n* = 401). Using functional metabolomics strategies, we found that six anti‐inflammatory/pro‐resolving oxylipin combinations had synergistic and cardioprotective effects on a murine model of myocardial ischemia‒reperfusion injury. Mechanistically, we demonstrated that inflammatory/pro‐resolving oxylipins could reinstate the synthesis of ceramides and lysophosphatidylcholines and ameliorate inflammatory responses. Collectively, our studies highlight the great potential of bioactive oxylipins in prognostic prediction and therapeutics after STEMI.

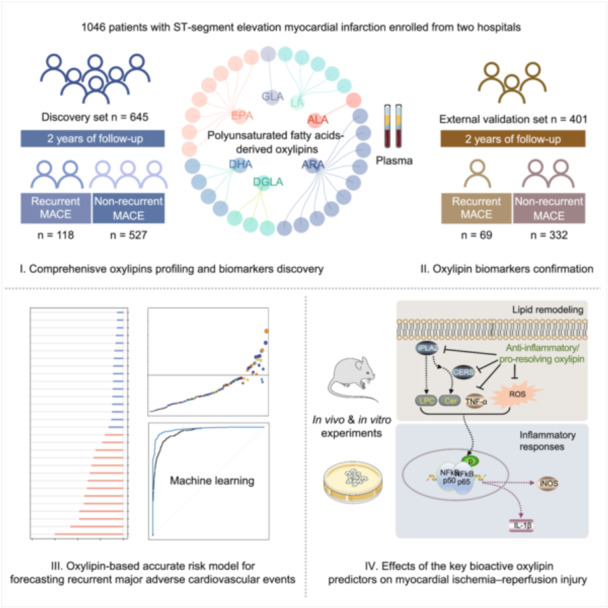

## INTRODUCTION

ST‐segment elevation myocardial infarction (STEMI) is the most severe and acute manifestation of coronary artery disease with a major cause of morbidity and mortality worldwide [1]. In the last two decades, advanced progress in early revascularization and secondary prevention has dramatically decreased the mortality of patients with STEMI [2, 3]. Despite the implementation of timely reperfusion therapy and stringent guideline‐based secondary prevention management (e.g., anticoagulation, antiplatelet, and angiotensin‐converting enzyme inhibitors), a substantial proportion of patients with STEMI remain at high risk for experiencing short‐ or long‐term adverse cardiovascular outcomes [4, 5].

Previous myocardial infarction (MI) events are the most important risk factor for the recurrence of major adverse cardiovascular events (MACE) in the STEMI population [6]. Although there are a variety of residual risk factors and prognostic assessments that are being targeted by advanced therapies, the long‐term mortality and recurrent MACE rates after STEMI (e.g., heart failure and cardiac death) remain unacceptably high [6, 7]. Therefore, there is still a great need to understand the exact mechanisms by which pathophysiological progression contributes to adverse cardiovascular outcomes and discover novel risk markers for prognostic management after STEMI.

In the past several decades, a growing body of experimental and clinical evidence has suggested a strong association between inflammation and recurrent adverse cardiovascular events in patients with STEMI [8]. A variety of novel pro‐inflammatory markers and therapeutic strategies that target inflammation have shown potential for reducing the risk of recurrent MACE events [9, 10]. Despite an evolving understanding of the inflammatory processes involved in the progression of STEMI, successful clinical translation of anti‐inflammatory therapies has proven challenging [11]. The molecular mechanisms underlying the inflammatory responses in the acute and reparative phases of STEMI are complex and remain largely unknown.

Oxylipins are a group of bioactive metabolites generated via enzymatic or nonenzymatic oxygenation of polyunsaturated fatty acids (PUFAs) that are involved in a variety of pathophysiological conditions, such as inflammation, oxidative stress, thrombosis, and atherosclerosis [12, 13, 14]. The enzymatic oxidation of oxylipins is carried out by three major enzyme systems, namely cyclooxygenases (COXs), lipoxygenases (LOXs), and cytochrome P450s (CYP450s). Oxylipins derived from different PUFAs or oxidase enzymes have been found to show distinct effects, for example, pro‐inflammatory hydroxy‐eicosatetraenoic acid (HETE) and anti‐inflammatory epoxy‐eicosatrienoic acid (EET) [15, 16]. Recent evidence has demonstrated that a variety of bioactive oxylipins might be novel prognostic markers and therapeutic targets for inflammatory and cardiovascular diseases, such as myocardial ischemia‒reperfusion (MI/R) and heart failure [15, 16, 17, 18].

To date, limited clinical data exist on the prognostic value of oxylipins in the STEMI population. In this study, we aimed to comprehensively investigate the association of circulating oxylipin profiles with recurrent MACE and to explore an oxylipin‐based risk model for secondary prevention in a large cohort of STEMI patients (*n* = 985). The findings were subsequently validated in an independent and external STEMI cohort (*n* = 562). We also sought to investigate the roles of the key oxylipin predictors in a murine MI/R model via functional metabolomic strategies.

## RESULTS

### Baseline clinical characteristics of the study populations

**Figure 1:**
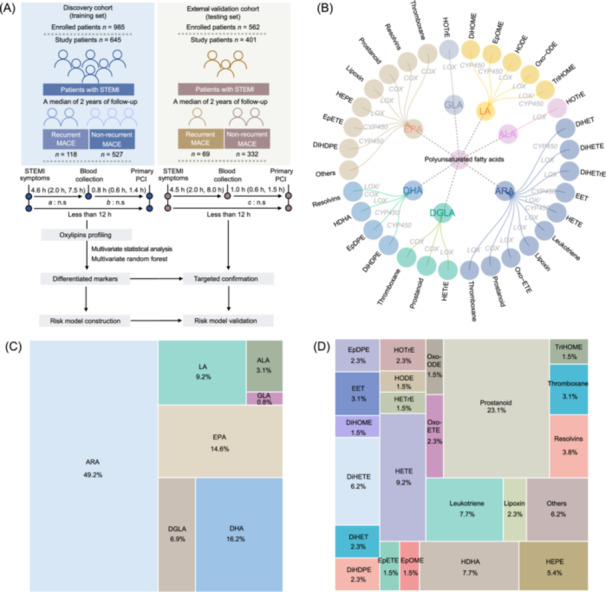
Clinical study design and comprehensive oxylipin profiles. (A) Cohort size, sample collection, and experimental overview. The duration is expressed as the median and interquartile range. n.s: No statistical differences in blood collection timepoints between the discovery and validation cohorts. *a*, duration between the onset of ST‐segment elevation myocardial infarction (STEMI) symptoms and the time of blood collection; *b*, duration between the time of blood collection and the start time of primary percutaneous coronary intervention (PCI); *c*, duration between the onset of STEMI symptoms and the start time of primary PCI. (B) Circle plot of polyunsaturated fatty acid (PUFA)‐derived oxylipins and the main metabolic genes/enzymes involved in oxidative generation signaling. (C, D) Treemap overview of the category distribution of all detected oxylipins in the discovery cohort based on the prototype PUFA precursors and their derived oxylipin classes, respectively. ARA, arachidonic acid; ALA, alpha‐linolenic acid; DHA, docosahexaenoic acid; DGLA, dihomo‐γ‐linolenic acid; DiHDPE, dihydroxy‐docosapentaenoic acid; DiHET, dihydroxy‐eicosatrienoic acid; DiHETE, dihydroxy‐eicosatetraenoic acid; DiHOME, dihydroxy‐octadecenoic acid; EET, epoxy‐eicosatrienoic acid; EpDPE, epoxy‐docosapentaenoic acid; EPA, eicosapentaenoic acid; EpETE, epoxy‐eicosatetraenoic acid; EpOME, epoxy‐octadecenoic acid; GLA, gamma linolenic acid; HDHA, hydroxy‐docosahexaenoic acid; HEDE, hydroxy‐eicosadienoic acid; HEPE, hydroxy‐eicosapentaenoic acid; HETE, hydroxy‐eicosatetraenoic acid; HETrE, hydroxy‐eicosatrienoic acid; HODE, hydroxy‐octadecadienoic acid; HOTrE, hydroxy‐octadecatrienoic acid; HpODE, hydroxy‐octadecatrienoic acid; LA, linoleic acid; oxo‐EET, oxo‐eicosatetraenoic acid; MACE, major adverse cardiovascular events; oxoODE, oxo‐octadecadienoic acid.

After the application of strict exclusion criteria, a total of 645 subjects from 985 adult STEMI patients who were enrolled at Beijing Anzhen Hospital were included in the discovery cohort. Another 401 individuals from the Peking University Third Hospital‐built external cohort consisting of 562 STEMI patients were included as the independent validation set (Figure 1A). All plasma samples in both cohorts were collected before primary percutaneous coronary intervention (PCI). The baseline characteristics of the enrolled individuals are provided in Table S1. In the discovery cohort, the patient population was predominantly male (66.8%), with a median age of 53.2 years, and 5.6% had a history of MI. During a median follow‐up of 2 (1.5–3.0) years, 118 patients (18.3%) experienced recurrent MACE. Among the 401 STEMI patients (male, 64.3%) included in the external validation cohort, 69 individuals (17.2%) experienced recurrent MACE during follow‐up. Furthermore, in both cohorts, no significant differences were found in the common cardiovascular risk factors (such as the occurrence of hypertension, diabetes, hypercholesterolemia, or myocardial injury markers) between the recurrent MACE and non‐recurrent MACE groups.

### Patients with and without recurrent MACE in the discovery cohort exhibited significant differences in various oxylipin subclasses

**Figure 2:**
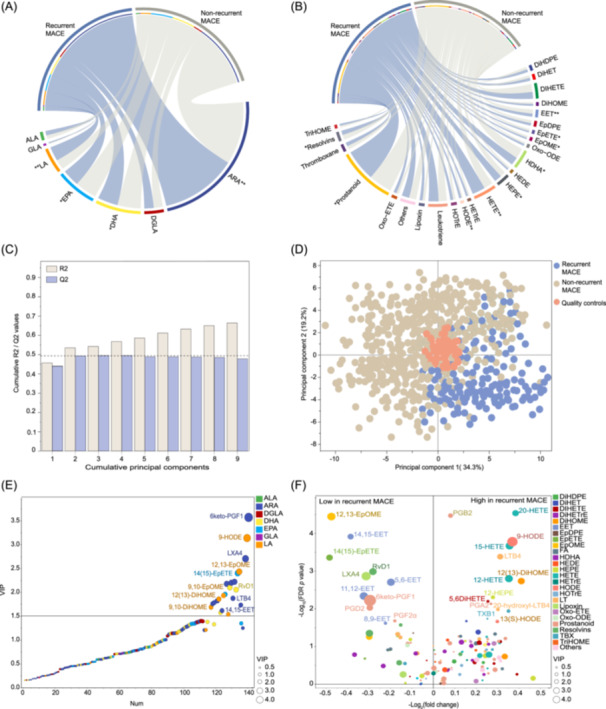
Pattern analysis of the oxylipin profiles between the recurrent MACE and non‐recurrent MACE groups in the discovery cohort. Chord diagrams of the differentiated oxylipin categories based on the prototype precursors (A) and oxidative derivatives (B). The width of the curves indicates the mean percentage of each oxylipin category between the recurrent and non‐recurrent MACE groups; * or ** indicates a *p*‐value < 0.05 or <0.01. (C) Cumulative Q2 and R2 value plots for selecting the optimized number of principal components in the establishment of principal component analysis (PCA) model. (D) PCA score plot of the plasma oxylipin profiling datasets from patients with and without recurrent MACE events. (E) Partial least square discriminant analysis‐derived variable importance projection (VIP) plot. The whole set of detected oxylipins are ranked according to their VIP values, and the circle size is proportional to the VIP value. A VIP value >1.5 represents the significant importance of the oxylipin individual in differentiating recurrent MACE and non‐recurrent MACE groups. (F) Integrated volcano plot of oxylipin variations in pairwise comparisons of recurrent MACE and non‐recurrent MACE groups. The significance thresholds for one‐dimensional differences were FDR‐adjusted *p* < 0.05, fold change >1.3. TXB1, thromboxane B1; 11‐keto‐TXB2, 11‐keto‐thromboxane B2; 6 keto‐PGF1, 6 keto‐prostaglandin F1; RvD1, resolvin D1; LXA4, lipoxin A4; LTB4, leukotriene B4.

First, we systematically profiled the changes in seven prototype PUFA precursors and 130 species of oxylipin products metabolized by COX, LOX and CYP450 enzymes in the plasma samples of the discovery cohort (Figure 1B). The detailed native oxylipin standards used for oxylipin profiling and the relative standard deviation values for all deuterated oxylipin standards throughout the experiments are shown in Tables S2, S3, respectively. The detected oxylipins mainly originated from four PUFAs (Figure 1C), including arachidonic acid (ARA, 49.2%), eicosapentaenoic acid (EPA, 14.6%), docosahexaenoic acid (DHA, 16.2%), and linoleic acid (LA, 9.2%). The 22 oxylipin subclasses and their proportional distributions are depicted in Figure 1D. On the basis of univariate analysis and chord diagrams (statistical threshold, *p* < 0.05), we found that the differentiated oxylipins between the recurrent MACE and non‐recurrent MACE groups were mainly derived from ARA, LA, and EPA (Figure 2A), including nine oxylipin subclasses (Figure 2B), namely HETE, EET, prostanoid, hydroxy‐octadecadienoic acid (HODE), hydroxy‐eicosapentaenoic acid, epoxy‐eicosatetraenoic acid (EpETE), epoxy‐octadecenoic acid (EpOME), resolvins, and hydroxy‐docosahexaenoic acid.

### Multivariate/univariate statistical analysis identified a variety of oxylipin species that were associated with recurrent MACE in the discovery cohort

Based on the datasets of PUFA and oxylipin profiling from the discovery set, we employed unsupervised principal component analysis (PCA) to explore the potential group separation between the recurrent MACE and non‐recurrent MACE groups. The first two principal components with satisfactory cumulative R2 (0.535) and Q2 (0.493) values were selected for constructing the PCA model (Figure 2C). As shown in Figure 2D, the PCA score plot showed that patients with recurrent MACE were remarkably distinguished from non‐recurrent MACE patients, suggesting a differentiated oxylipin landscape between the two study groups. In the beta‐coefficient regression analysis, the correlation plots indicated that most of the detected oxylipins had few associations with commonly known risk factors, such as age, hypertension, diabetes, and myocardial injury markers (Figures S1–S5).

We then integrated a supervised partial least square discriminant analysis (PLS‐DA)‐based variable importance projection (VIP) plot and univariate analyses to identify the differentiated oxylipins contributing to the class separation between the recurrent MACE and non‐recurrent MACE groups. Twenty‐two oxylipins with a significant VIP value ≥1.5 were identified, and those differentiated oxylipins were mainly derived from the oxidative metabolism of ARA and LA (Figure 2E). Compared with patients in the non‐recurrent MACE group, individuals in the recurrent MACE group presented increased levels of leukotriene (LT) B4, 20‐hydroxyl‐LTB4, prostaglandin (PG) A2, PGB2, thromboxane B1 (TXB1), 9‐HODE, 13(S)‐HODE, and several HETEs and decreased levels of 12,13‐EpOME, 14(15)‐EpETE, PGD2, PGF2α, 6keto‐PGF1, four EETs, and two resolvins (Figure 2F).

### The random forest algorithm identified a 14 oxylipin marker‐based risk model that showed remarkable performance in predicting recurrent MACE in the discovery cohort

**Figure 3:**
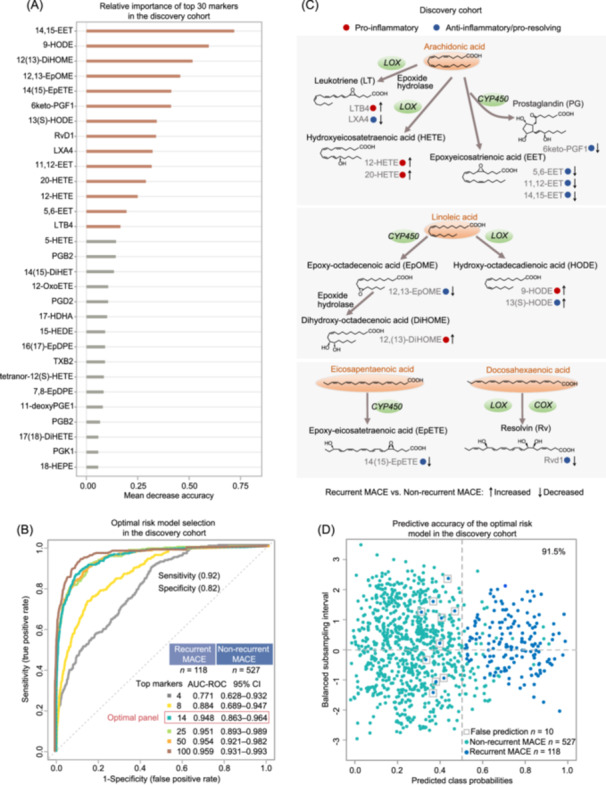
Oxylipin marker panel selection and risk model construction for predicting recurrent MACE in the discovery cohort. (A) The top 30 important recurrent MACE‐associated oxylipins ranked by the mean decrease accuracy values in the adjusted‐multivariate random forest risk model. The adjusted‐covariates included age, sex, aspirin uses, previous PCI, hypertension, diabetes, body mass index, troponin T, troponin I, hypersensitive C‐reactive protein, and brain natriuretic peptide. (B) Receiver operating curve (ROC) generated from Monte Carlo cross‐validation‐based multivariate random forest models using different numbers of top oxylipin markers. The values of the area under the ROC curves (AUC‐ROC), specificity, sensitivity, and 95% confidence interval (CI) are shown. (C) The expression trends, major biosynthetic pathways, and bioactive properties of the top‐14 oxylipin predictors in the discovery cohort. (D) Posterior classification probability plot based on the random forest model using the top‐14 oxylipins in the discovery cohort. PGK1, prostaglandin K1; 14(15)‐DiHET, 14,15‐dihydroxy‐eicosatrienoic acid; 11‐deoxy PGE1, 11‐deoxy prostaglandin E1.

An ensemble‐supervised random forest (RF) algorithm was used to select potential risk markers and construct risk models for predicting recurrent MACE in patients with STEMI from the discovery cohort. Each detected PUFA precursor and oxylipin derivative were ranked by variable importance. The top 30 recurrent MACE‐associated markers obtained from the unadjusted‐ or adjusted‐ RF algorithm are summarized in Figure 3A and Figure S6A, B. Notably, combined adjustments for age, sex, aspirin use history, and clinical risk factors demonstrated few effects on the ranking order and mean decrease accuracy values of the top 30 markers. Then, we used multivariable RF model‐based Monte Carlo cross‐validation analysis to assess the predictive performance of different numbers of top important variables and select the optimal marker panel. As shown in Figure 3B, the optimal panel consisting of the top 14 features showed significant performance in predicting recurrent MACE with higher area under receiver operating curve (AUC‐ROC) values, whereas the additional variables had little effect on the AUC‐ROC values.

The bioactive properties, prototype PUFAs, and synthetic pathways of these 14 oxylipin markers are depicted in Figure 3C. Compared with the non‐recurrent MACE group, the recurrent MACE group expressed decreased levels of eight anti‐inflammatory/pro‐resolving oxylipins [14,15‐EET, 12,13‐EpOME, 14(15)‐EpETE, 6keto‐PGF1, resolvin D1 (RvD1), lipoxin A4 (LXA4), 11,12‐EET, and 5,6‐EET]. Additionally, the plasma levels of one anti‐inflammatory oxylipin [13(S)‐HODE] and five pro‐inflammatory oxylipins [9‐HODE, 12(13)‐dihydroxyoctadec‐12‐enoic acid (DiHOME), 20‐HETE, 12‐HETE, and leukotriene B4 (LTB4)] were found to be higher in patients with recurrent MACE than those in the patients without recurrent MACE. The posterior classification probability plot of these 14 oxylipin markers revealed a high correct prediction rate (108 in 118) and significant predictive accuracy (91.5%) for predicting recurrent MACE (Figure 3D). The reliability of the established RF model was also confirmed by the permutation test (*p*‐value < 0.0001).

### The predictive value of the oxylipin marker panel from the discovery cohort was confirmed in an independent external validation cohort

**Figure 4:**
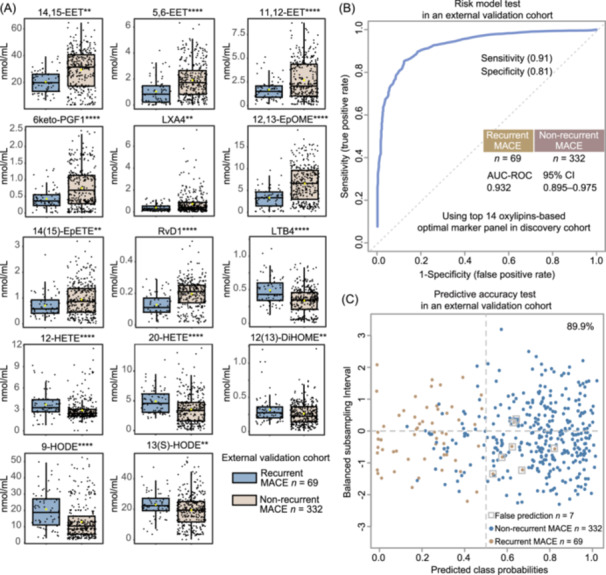
Validation of the discriminatory ability of the 14 risk oxylipin predictor panel in an independent external cohort. (A) Boxplot of the expression levels of the 14 recurrent MACE‐related oxylipin signatures in the external validation cohort. The Mann‒Whitney *U* test was used for each comparison (** and **** indicate *p* values < 0.01 and *p* values < 0.0001, respectively). (B) The predictive performance of the 14 risk oxylipin panel in the external cohort using the multivariate random forest‐based ROC curve. The AUC‐ROC value, specificity, sensitivity, and 95% CI are shown. (C) Posterior classification probability plot for evaluating the predictive accuracy of the 14‐oxylipin risk panel in the external cohort.

To test the prognostic value of the 14‐oxylipin marker panel for predicting the risk of recurrent MACE, targeted metabolomics analysis was performed on plasma samples collected from the external validation cohort. The quantitative levels of 14 oxylipin markers between the recurrent MACE and non‐recurrent MACE groups in the validation cohort are summarized in Figure 4A. Our results also demonstrated that the expression trends of all 14 markers between recurrent MACE and non‐recurrent MACE were consistent in the discovery and validation cohorts (Figures 3C and 4A). Based on the multivariate RF‐based risk model from discovery cohort, the 14‐oxylipin markers also exhibited a significant discriminatory ability (AUC‐ROC value = 0.932) in the external validation cohort (Figure 4B). As shown in Figure 4C, the predictive accuracy of the 14 oxylipin panel‐based risk model in the external validation cohort was also highlighted with a significantly accurate prediction rate (89.9%). Furthermore, all these 14 oxylipin markers showed few associations with aspirin use in the discovery and validation cohorts (Figure S6C).

### Anti‐inflammatory/pro‐resolving oxylipins exhibited greater prognostic values than pro‐inflammatory oxylipins

**Figure 5:**
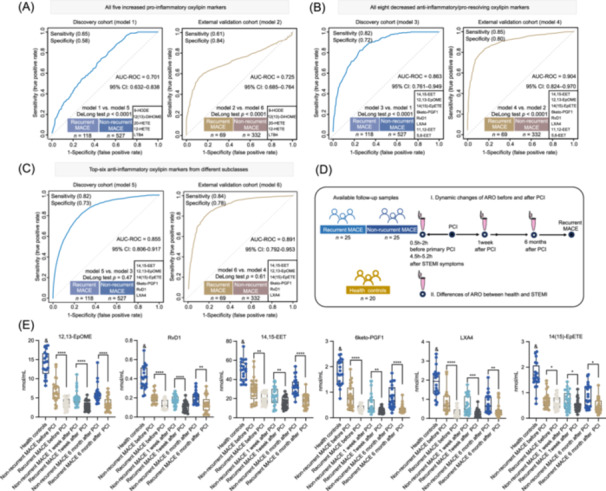
Prognostic assessments of pro‐inflammatory and anti‐inflammatory/pro‐resolving oxylipin markers for predicting recurrent MACE in the discovery and validation cohorts. (A–C) ROC curves generated from the Monte Carlo cross validation‐based multivariate random forest models using the datasets of all five increased pro‐inflammatory (PO) markers (A, model 1 and model 2), all eight decreased anti‐inflammatory/pro‐resolving oxylipin (ARO) markers (B, model 3 and model 4), top‐six ARO predictors (C, model 5 and model 6). The AUC‐ROC values, specificity, sensitivity, and 95% CI are shown. A DeLong test *p* < 0.05 indicated a significant difference in the comparison of ROC‐AUC values from different models. (D) Study design for exploring the changes in the plasma samples of STEMI patients after PCI and determining the plasma levels of six ARO predictors in healthy individuals. (E) Dot histogram of the six ARO metabolites in plasma samples at different timepoints after PCI in patients with STEMI and the plasma samples of healthy individuals. The Mann‒Whitney *U* test was used for each comparison (*, **, *** or **** indicate *p* values < 0.05, *p* values < 0.01, *p* values < 0.001, or *p* values < 0.0001, respectively). ^&^ indicates a *p‐*value < 0.01 when health controls were compared with STEMI patients at different follow‐up timepoints.

To explore the prognostic values of anti‐inflammatory/pro‐resolving oxylipin (ARO) and pro‐inflammatory oxylipin (PO), the five increased PO combination and eight decreased ARO combination in the 14‐marker panel were used to construct RF risk modes in both the discovery and external validation cohorts. Impressively, the top‐eight AROs combination showed better performance in predicting recurrent MACE than the five‐PO combination in both cohorts, as evidenced by the ROC‐AUC values and DeLong test *p* values (Figure 5A,B). The top‐six ARO predictors, including 14,15‐EET, 12,13‐EpOME, 14(15)‐EpETE, 6keto‐PGF1, RvD1, and LXA4 are derived from different oxidative pathways of ARA, LA, EPA, and DHA and belong to six oxylipin subclasses (Figure 3C). Then, we explored the predictive performances of the top‐six ARO combination. Notably, the results indicated that the top‐six ARO combination also exhibited greater prediction performances than the five‐PO combination (Figure 5A, DeLong test *p* values < 0.0001 in the discovery and validation cohorts). There were no statistical differences in the ROC‐AUC values between top‐six ARO‐based risk model and top‐seven/top‐eight ARO‐based risk models in both cohorts (Figure 5C and Figure S7, DeLong test *p* values > 0.05). However, our results demonstrated that the top‐six ARO combination showed better predictive performances in differentiating recurrent MACE and non‐recurrent MACE than the other top‐ARO combination, as evidenced by the DeLong test (Figure S7, all *p* values < 0.001 in the discovery and validation cohorts).

### STEMI patients with recurrent MACE expressed lower plasma ARO levels than subjects without recurrent MACE during the follow‐up period

To further investigate the changes in the plasma levels of top‐six AROs during the follow‐up period after primary PCI, the targeted analysis was performed on plasma samples before primary PCI, 1 week after primary PCI, and 6 months after primary PCI in a total of 50 matched STEMI patients with and without recurrent MACE (Figure 5D). The baseline characteristics of the enrolled individuals are provided in Table S4. As shown in Figure 5E, the plasma levels of six AROs at 1 week and 6 months after PCI were also lower in patients who experienced recurrent MACE than in individuals without recurrent MACE (*p* value < 0.05). Furthermore, we also determined the differences in plasma ARO levels between STEMI patients and healthy individuals (Figure 5D). Our results indicated that the plasma levels of six AROs in healthy individuals (*n* = 20) were higher than those in STEMI patients with and without recurrent MACE (Figure 5E; *p*‐value < 0.01), suggesting that AROs might be consumed during the progressive phases of STEMI.

### Combined treatment of ARO exhibited significant cardiac effects on MI/R model mice compared with the combined treatment of PO and the individual ARO treatment

**Figure 6:**
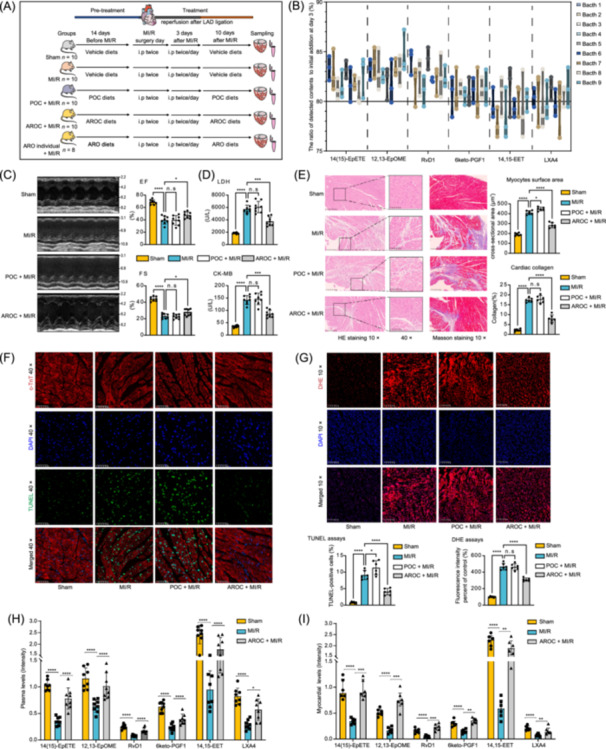
Evaluation of the in vivo effects of pro‐inflammatory oxylipins and anti‐inflammatory/pro‐resolving oxylipins on myocardial ischemia‒reperfusion model mice. (A) Experimental animal study overview. Vehicle diets, pro‐inflammatory oxylipin combination (POC)‐containing diets, individual ARO‐containing diets, and ARO combination (AROC)‐containing diets were added with antioxidants. (B) The ratio of ARO contents on day three compared with the initial addition amounts in nine batches of mouse chow‐containing AROC (*n* = 3 feed‐pellets in each batch). (C) Representative images of echocardiography and quantitative results (*n* = 8 mice in each group). (D) Plasma levels of myocardial injury markers (*n* = 8 mice in each group), including creatine kinase (CK)‐MB and lactate dehydrogenase (LDH). (E) Histological analysis of heart tissues stained with hematoxylin‐eosin (HE) to demarcate myocyte surface areas and Masson's trichrome to determine collagen deposition (*n* = 6 regions in each group). (F) TdT‐mediated dUTP nick‐end labeling (TUNEL)‐positive and cardiac troponin (c‐TnT) double‐stained cardiomyocytes (*n* = 6 regions in each group). (G) Dihydroethidium (DHE)‐labeled reactive oxygen species staining of heart tissues (*n* = 6 regions in each group). (H, I) The plasma and myocardial concentrations of six AROs after four weeks of continuous AROC treatment (*n* = 6 or 8 mice in each group). *, **, *** or **** indicate *p* values < 0.05, *p* values < 0.01, *p* values < 0.001, or *p* values < 0.0001, respectively. MI/R, myocardial ischemia‒reperfusion; LAD, left anterior descending coronary artery; i.p, intraperitoneal injection; EF, ejection fraction; FS, fractional shortening.

To determine whether the ARO or PO predictors have protective or cardiotoxic effects on post‐reperfusion injury after PCI, we used an MI/R murine model that mimics human STEMI after PCI. Based on the prognostic values of different combinations of AROs and POs (Figure 5A–C), the five PO combination (POC), the top‐six ARO combination (AROC), and six individual ARO were selected for animal studies. The entire procedure of the animal study design is shown in Figure 6A. To maintain a steady‐state concentration of oxylipins in mice, the administration of oxylipins was mainly depended on daily dietary supplementation. Because oxylipins are unstable, we used antioxidant additives to maintain the stability of oxylipins in mouse chow. We also determined the changes of ARO and PO in the chow under different light conditions, and the results indicated that the residual ratios of oxylipins in the chow on day 3 were >70% for 24 h‐light conditions and >85% for 24 h‐dark conditions, and extended storage time has resulted in a large amount of oxylipin loss (Figure S8). Therefore, the feed was renewed every 3 days during the experiment.

The optimal administered dose of each oxylipin in the AROC and POC was calculated on the basis of the fold changes in patients with and without recurrent MACE (Figure S9A, B). The residual ratios of oxylipins in the nine batches of chow containing AROC, POC, and individual ARO during the animal experiments were greater than 75%–80% (Figure 6B and Figure S9C, D). After treatment, we found that POC did not significantly alter cardiac function and myocardial injury markers in MI/R mice, whereas AROC significantly protected against acute MI/R‐induced abnormalities in cardiac function and enzyme markers (Figure 6C,D). Our results also revealed that AROC significantly decreased cardiomyocyte hypertrophy, fibrotic remodeling, myocardial apoptosis, and reactive oxygen species accumulation, whereas POC only slightly promoted collagen deposition and myocardial apoptosis (Figure 6E–G). Furthermore, we also found that individual ARO treatment did not significantly improve cardiac function and myocardial injury markers (Figure S10A, B), and had few or slight effects on improving MI/R injury‐induced pathological changes in heart tissues (Figure S10C–F).

### AROC treatment significantly elevated the plasma and myocardial concentrations of ARO in MI/R mice

Next, we performed targeted quantitative metabolomics to analyze the changes in the plasma and myocardial levels of six AROs after AROC treatment. The results indicated that the plasma levels of the six AROs in the MI/R group were significantly lower than those in the Sham group (Figure 6H), which is in line with our findings in humans (Figure 5E). In addition, we observed that the myocardial levels of AROs were markedly lower in the MI/R group than those in the Sham group (Figure 6I). After four consecutive weeks of AROC treatment, the levels of six AROs in the plasma and heart tissues were significantly increased in MI/R mice (*p* values < 0.05; Figure 6H,I). Collectively, these results demonstrated that the cardioprotective actions of AROC treatment might be directly mediated by the increased in vivo ARO exposures.

### AROC exhibited synergistic effects on improving myocardial metabolism remodeling after MI/R

**Figure 7:**
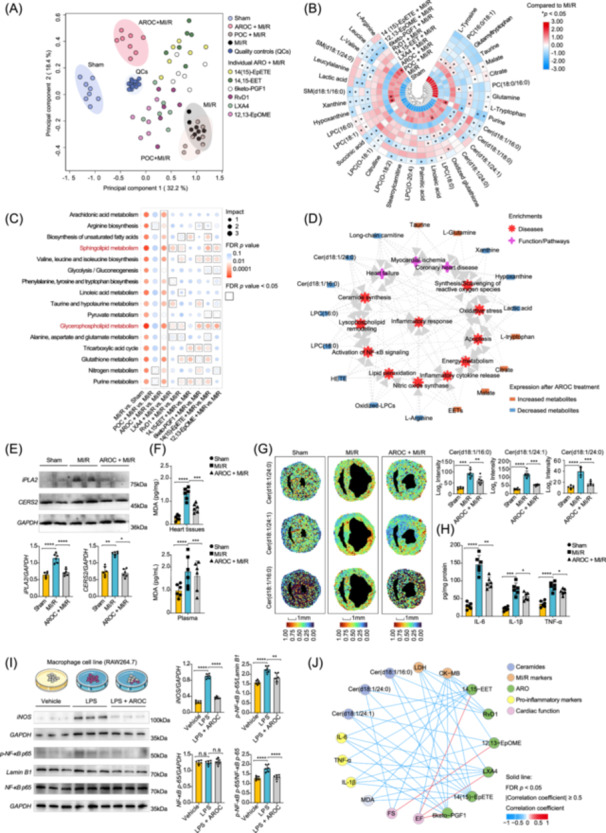
Effects of anti‐inflammatory/pro‐resolving oxylipins on regulating myocardial ischemia‒reperfusion injury‐induced perturbations in myocardial metabolism and inflammatory signaling. (A) PCA score plot of the myocardial metabolome from the POC‐, individual ARO‐, and AROC‐treated groups (*n* = 8 mice in POC‐ and AROC‐treated group; *n* = 6 mice in individual ARO‐treated groups). (B) Heatmap showing the effects of AROC, POC, and individual ARO on myocardial metabolome disturbances in MI/R model mice. (C) Metabolic pathway enrichment plot of differentiated metabolites in different group comparisons. (D) Ingenuity pathway analysis‐based functional network for the AROC‐altered metabolites and associated biological pathways/diseases. Edges represent associations. (E) Representative western blot images of calcium‐independent phospholipase A2 (*iPLA2*) and ceramide synthase 2 (*CERS2*) in heart tissues and their quantification (*n* = 6 mice in each group). (F) Cardiac and plasma detection of malondialdehyde (*n* = 6 mice in each group). (G) Mass spectrometry imaging‐based myocardial distributions of ceramide (Cer) after AROC treatment (*n* = 6 regions in each group). (H) Myocardial levels of pro‐inflammatory cytokines using enzyme‐linked immunosorbent assays (*n* = 6 mice in each group). (I) Effects of AROC on nuclear factor‐kappa B (*NF‐κB*) activation and inducible nitric oxide synthase (*iNOS*) expression in lipopolysaccharide (LPS)‐induced pro‐inflammatory M1 macrophages (*n* = 6 cellular experiments in each group). *, **, *** or **** indicate *p* values < 0.05, *p* values < 0.01, *p* values < 0.001, or *p* values < 0.0001, respectively. (J) Spearman correlation between AROs and MI/R‐induced biochemical and cardio‐functional alterations. The correlations with absolute coefficients ≥ 0.5 and FDR‐adjusted *p*‐value < 0.05 are shown as solid lines. IL‐1β, interleukin‐1beta; IL‐6, interleukin‐6; LPC, lysophosphatidylcholine; MDA, malondialdehyde; PC, phosphatidylcholine; TNF‐α, tumor necrosis factor‐alpha.

We subsequently performed untargeted metabolomics to investigate the potential effects of AROC, POC, and six individual AROs on the myocardial metabolism of MI/R model mice. The categories of the identified metabolites are summarized in Figure S11A. The PCA score plot derived from the metabolomic data revealed a clear separation trend between the MI/R group and Sham group, whereas the AROC + MI/R group remarkably deviated from the MI/R group and was located close to the Sham group (Figure 7A). The six individual ARO‐treated groups presented different degrees of deviation from the MI/R group, but their effects on improving the overall metabolome profile were inferior to those of AROC. However, we did not observe clear group separation between the POC + MI/R group and the MI/R group. By using the volcano plot (Figure S11B), the differentiated metabolites that contributed to the group separation among the MI/R, Sham, and AROC‐treated groups were identified, including a variety of amino acids and derivatives, phospholipids, carbohydrates, sphingolipids and fatty acyls. Importantly, our results demonstrated that different types of ARO showed various effects on the AROC‐altered metabolites and associated metabolic pathways (Figure 7B,C). These results suggested that different AROs in AROC might have synergistic effects on improving metabolic remodeling after MI/R. However, POC treatment showed slight effects on the metabolomic alterations of MI/R model mice.

### AROC inhibited cardiac lysophospholipid remodeling and ceramide synthesis

The above metabolomic findings (Figure 7B–D) indicated an important role of AROC in regulating the metabolism of glycerophospholipids (lysophospholipid remodeling) and sphingolipids (ceramide synthesis). The levels of several lysophosphatidylcholines (LPCs) and oxidized LPCs were elevated after MI/R injury but significantly decreased after AROC treatment. Mechanistically, we found that the protein level of calcium‐independent phospholipase A2 (*iPLA2*), the upstream regulatory enzyme that regulates the release of LPC from phosphatidylcholines was decreased after AROC treatment (Figure 7E, *p* values < 0.05). Furthermore, the plasma and myocardial levels of malondialdehyde (the oxidizing marker for the peroxidation of LPC) were also found to be decreased (Figure 7F).

For alterations in sphingolipid metabolism, our results demonstrated that AROC could decrease the levels of five sphingolipid metabolism‐derived metabolites, particularly three lipotoxic ceramides (Cer, d18:1/16:0; d18:1/24:1; d18:1/16:0) in MI/R mice. Using targeted metabolomics, we confirmed that the three Cers were significantly lower in the plasma of ARO + MI/R group than those in the MI/R group (Figure S11C, all *p* values < 0.05). Moreover, we found that the administration of AROC significantly decreased the spatial distribution of the three ceramides and the protein levels of ceramide synthase 2 (*CERS2*) in heart tissues of MI/R model mice (Figure 7E,G, *p* value < 0.05).

### AROC ameliorated metabolic disturbance‐associated inflammatory responses in MI/R model mice

In the function/disease enrichment network of the ingenuity pathway analysis (Figure 7D), we found that LPCs, ceramides, and several other AROC‐altered metabolites were closely associated with inflammatory responses, including inflammatory cytokine release, nuclear factor‐kappa B (NF‐κB) activation, and nitric oxide synthase (NOS). Therefore, we further evaluated the effects of AROC on these network‐enriched inflammatory signaling pathways. Our results revealed that AROC significantly decreased the levels of pro‐inflammatory cytokines, including tumor necrosis factor‐alpha, interleukin (IL)‐1β, and IL‐6, in both myocardial and plasma samples from MI/R model mice (Figure 7H and Figure S11D, *p* values < 0.05). Regarding the upstream regulatory pathways of inflammatory cytokine release, we found that AROC significantly decreased the protein levels of phospho‐NF‐κB p65 and inducible NOS (iNOS) in lipopolysaccharide (LPS)‐induced pro‐inflammatory M1 macrophages (Figure 7I).

## DISCUSSION

Over the last two decades, the short‐term mortality of patients with STEMI has markedly decreased by the implementation of PCI therapy [19]. However, previous clinical evidence demonstrated that no change in cardiac infarct severity was observed in patients with STEMI after primary PCI [2]. Progressive left ventricular remodeling and adverse ischemia‒reperfusion injury still develop after PCI despite the combined use of guideline‐directed secondary prevention therapies [7, 20]. Inflammation not only plays an important role in the initiation and progression of atherogenesis and plaque evolution but also plays a critical role in determining acute infarct size, subsequent reperfusion injury, and post‐adverse left ventricular remodeling [21], making it a potential therapeutic target for improving clinical outcomes after STEMI. Oxylipins play a significant role in regulating inflammation, and increasing lines of evidence point toward their importance in cardiovascular diseases [22, 23].

For the first time, we systematically profiled the changes in circulating PUFAs and their pro‐inflammatory/anti‐inflammatory derivatives in a large set of patients with and without recurrent MACE after STEMI. Notably, our results indicated that the differentiated oxylipin markers showed few correlations with commonly known clinical risk factors and exhibited relatively independent risk associations with recurrent MACE. Using an RF‐based machine learning algorithm, we identified a 14 oxylipin‐based prognostic model that exhibited significant performance in predicting recurrent adverse clinical outcomes in patients with STEMI after PCI during 2 years of follow‐up. The prognostic value of the established oxylipin‐based marker panel was also confirmed in an independent external STEMI cohort.

Unlike previously reported prognostic markers (e.g., troponin and N‐terminal pro‐B‐type natriuretic peptide) which cannot provide additional information on therapeutic targets, this study also systematically described the cardiovascular and metabolic activities of recurrent MACE‐related PO and ARO metabolites in a murine MI/R model. Since these active oxylipins are derived from different PUFAs or oxidase systems, supplementing their PUFA prototypes or interfering with their upstream synthetase signals is an indirect approach to assess their effects on MI/R injury. In this study, we used the dietary supplement method to directly evaluate the in vivo activities of the key recurrent MACE‐related oxylipins. We also used antioxidants and liquid chromatography/mass spectrometer (LC/MS)‐based dynamic tracking strategies to ensure that these unstable oxylipins could be reliably delivered to the mice.

Our results showed that the combined use of six different types of ARO predictors has great potential for improving reperfusion pathogenesis compared with individual ARO. The decreased plasma levels of these bioactive ARO metabolites in MI/R mice could be significantly elevated after AROC treatment, and their plasma levels were negatively correlated with the levels of MI/R injury markers, inflammatory cytokines, and toxic ceramides (Figure 7J). Furthermore, the AROC treatment also exhibited synergetic effects on MI/R‐induced metabolic remodeling. Our results found that 14(15)‐EpETE showed multiple impacts on the metabolism of hydrophilic metabolites, including amino acid metabolism and purine metabolism, whereas RvD1 exhibited a specific effect on the regulation of sphingolipid metabolism and fatty acid metabolism. 14(15)‐EpETE and 12,13‐EpOME exhibited exclusive roles in regulating glycolysis and pyruvate metabolism. These ARO predictors are produced by different oxygenated metabolism pathways of omega‐3 or omega‐6 PUFAs, which have been reported to have cardioprotective activities [22, 23, 24, 25, 26, 27, 28, 29, 30].

12,13‐EpOME is generated from LA by CYP 450s, which has been reported to have a protective role against hypoxia/reoxygenation injury [22], and exhibit significant in vivo effects on improving cardiac function against adverse cardiac remodeling [24]. RvD1 is a pro‐resolving and antiapoptotic lipid mediator derived from DHA by LOX or COX enzymes, and its deregulation can lead to chronic inflammation in cardiovascular systems [25, 26]. RvD1 can attenuate the infiltration of pro‐inflammatory M1 macrophages and the myocardial expression of inflammatory cytokines and ameliorate myocardial apoptosis, hypertrophy and fibrosis by inhibiting the activation of NF‐κB signaling [27, 28]. EETs are oxygenated metabolites of AA that are generated by CYP450s and have been shown to have anti‐inflammatory and cardioprotective effects [29, 30]. Previous evidence has demonstrated that EETs, particularly 14,15‐EET, can suppress inflammatory molecule expression and the cardiac inflammatory response by attenuating the activation of NF‐κB and iNOS and preventing macrophage activation [31, 32].

At the metabolomic level, our results revealed that different types of ARO in AROC could synergistically relieve acute MI reperfusion‐induced disturbances in myocardial metabolism that involve a variety of metabolic pathways and functional signals. In particular, AROC played a significant role in regulating sphingolipid and glycerophospholipid metabolism, as evidenced by a significant decrease in the myocardial levels of ceramides and LPC. Ceramides and LPC are toxic lipids that play important roles in the development of cardiovascular diseases by regulating multiple signals of inflammation, atherosclerosis, oxidative stress, and apoptosis [33, 34]. Mechanistically, our results indicated that AROC treatment significantly decreased the protein expression levels of the upstream metabolic genes of ceramides and LPC, including *iPLA2* and *CERS2*. Furthermore, we also revealed that AROC could ameliorate ceramide‐ and LPC‐trigged the downstream signaling activation, as evidenced by the remarkable inhibition of NF‐κB and iNOS in LPS‐induced pro‐inflammatory macrophages.

Our study also demonstrated that a variety of POs were differentially expressed between recurrent MACE and non‐recurrent MACE. For instance, three HETE species (12‐, 15‐, 20‐) produced from ARA by LOX or CYP450 enzymes were significantly elevated in patients with recurrent MACE. 20‐HETE and the two mid‐chain HETEs (12‐ and 15‐) have been linked to vascular dysfunction, myocardial fibrosis, hypertrophy, and dysfunction due to their strong effects on immune cells, cardiomyocytes, and fibroblasts via the activation of multiple receptors that lead to the activation of inflammatory cascades [35, 36, 37]. We also observed that pro‐inflammatory 9‐HODE was increased in plasma samples from patients with recurrent MACE. 9‐HODE is derived from LA by LOX and CYP epoxygenases, which can induce oxidative stress and inflammation by activating endoplasmic reticulum stress and the secretion of inflammatory cytokines [38, 39]. Unexpectedly, our results revealed that the combined treatment of five important POs in the risk marker panel slightly aggravated post‐acute myocardial injury and metabolic remodeling in MI/R model mice. Further studies designed to explore the effects of PO deprivation on MI/R might help us to comprehensively understand the pathophysiological effects of PO on STEMI.

Although this study highlighted novel evidence of the associations of bioactive oxylipins with adverse clinical outcomes after STEMI based on two independent cohorts with over 1000 STEMI patients. Some limitations warrant discussion. First, the ethnic homogeneity of the study population might limit the generalizability of our findings to other populations. Second, further studies designed to investigate the effects of lifestyle and environmental factors on oxylipin profiles may offer great promise for exploring additional intervention strategies to combat adverse clinical outcomes after STEMI. Third, in‐depth studies on the specific downstream targets of the bioactive oxylipin may help us to comprehensively understand the oxylipin‐mediated actions underlying adverse outcomes after STEMI.

## CONCLUSION

In conclusion, the present study demonstrated that a variety of PUFA‐derived oxylipin metabolites serve as residual risk markers for prognosis after STEMI, and the oxylipin‐based risk model exhibited powerful performance in predicting recurrent MACE. Furthermore, this study opens a new range of possibilities for the design of bioactive oxylipin agents as intervention points to mitigate post‐MI/R pathogenesis with significant therapeutic potential for improving adverse clinical outcomes after STEMI.

## METHODS

### Patients and blood sample collection

For the discovery cohort set, we enrolled 985 consecutive adult patients (>18 years of age) with STEMI between 2021 and 2023 at Beijing Anzhen Hospital. For the external validation cohort, 562 adult patients with STEMI were enrolled from Peking University Third Hospital between 2019 and 2023. STEMI was defined as characteristic symptoms lasting more than 30 min, with electrocardiographic ST‐segment elevation at least 0.1 mV in >2 contiguous limb leads or at least 0.2 mV in >2 contiguous precordial leads, >0.1 mV within 2 contiguous leads, and creatine kinase elevation more than the doubled normal upper limit. The exclusion criteria were as follows: patients with severe heart failure, serious heart valve disease, genetic or known cardiomyopathy, familial hypercholesterolemia, digestive diseases, current infectious diseases, chronic kidney diseases, pregnancy, or malignancies at baseline; and patients who were unable or unwilling to provide blood samples. After exclusion, a total of 1046 patients with STEMI from the two cohorts were enrolled in the metabolomics study. To explore the dynamic changes in the top‐six AROs during follow‐up, 50 aged‐ and sex‐matched STEMI patients were enrolled from the external validation cohort. Healthy controls were defined as subjects with no history of any cardiovascular diseases. The exclusion criteria for healthy controls were consistent with the above exclusion criteria for STEMI patients.

For all enrolled patients in both cohorts, blood samples were collected within 12 h after the onset of STEMI symptoms and 0.5–2 h before primary PCI‐based reperfusion therapy. The demographic characteristics, medical history, symptoms at admission, in‐hospital treatment and clinical outcomes were recorded. Clinical follow‐up information and outcomes were prospectively collected after 12 months and 24 months by reviewing the electronic patient records and by annual telephone calls, and these datasets were verified by medical practitioners. The primary endpoint was defined as recurrent MACE, which consisted of cardiac death, nonfatal myocardial infarction, repeat revascularization, and heart failure. This study complies with the Declaration of Helsinki and was approved by the Ethics Committee of Beijing Anzhen Hospital of the Capital University of Medical Sciences and Peking University Third Hospital. Verbal and written consent was obtained from all the subjects.

### Chemicals and reagents

LC/MS grade methanol, acetonitrile, and formic acid were purchased from Fisher Scientific. LC/MS grade methyl tert‐butyl ether (MTBE) was purchased from Sigma‐Aldrich. Anti‐calcium‐independent PLA2 (ab259950) and anti‐CERS2 (ab315452) antibodies were purchased from Abcam. Phospho‐NF‐ kappa B p65 (BY0127) and NF‐κB p65 (CY5034) antibodies were obtained from Abways. iNOS antibody (2226‐1‐AP) and enzyme‐linked immunosorbent assay (ELISA) kits for tumor necrosis factor‐α (KE10002), interleukin‐1β (KE10003), and interleukin‐6 (KE10007) were obtained from Proteintech. Cer (d18:1/16:0), Cer‐d_*7*_ (d18:1/16:0), Cer (d18:1/16:0), Cer‐d_*7*_ (d18:1/16:0), Cer (d18:1/24:1), Cer‐d_*7*_ (d18:1‐d7/24:1(15Z)), and all deuterated and native chemical standards of PUFA and oxylipins were obtained from Cayman Chemical, and the detailed list is depicted in Tables S2, S3.

### PUFA and oxylipin extraction and analysis

The extraction method for plasma PUFA and oxylipin was performed according to our recently published work [40]. The detailed extraction optimization is shown in the Supplementary information. Briefly, solid‐phase extraction (SPE) and methods that combine protein precipitation (PPT) with liquid‒liquid extraction (LLE) were compared during sample preparation. Finally, PPT (methanol with 1% formic acid as a protein precipitant) combined with LLE (MTBE/methanol, 7:3, v/v; 1% formic acid as an extractant) with satisfactory recovery and minimal matrix effects was chosen for the present study (Figure S12A–C). Additionally, the established oxylipin method showed high sensitivity for resolvin detection in the plasma samples (Figure S12D, E). The detailed protocol was as follows: Frozen plasma samples were thawed at 4°C, and 50 μL of each sample was precipitated by adding 200 μL of ice‐cold methanol with 1% formic acid containing the deuterated PUFA and oxylipin standards (the detailed concentrations are shown in Table S3). The mixture was vortexed for 2 min and centrifuged at 15,000 rpm for 15 min at 4°C, and the upper supernatants were collected as upper phase I. Subsequently, 200 μL of ice‐cold MTBE/methanol (7:3, v/v) with 1% formic acid was added to the residual precipitate. After vortex‐blending for 2 min, the mixture was centrifuged at 15,000 rpm for 15 min at 4°C. The upper supernatants were collected as upper phase II and combined with upper phase I. Before analysis, the eluent was dried under vacuum and redissolved in 100 μL of methanol‐acetonitrile (1:1, v/v) for LC/MS analysis.

The levels of PUFAs and their oxylipin derivatives in the plasma extract were detected via the AB Sciex QTRAP LC/MS platform of the State Key Laboratory of Natural and Biomimetic Drugs (https://sklnbd.bjmu.edu.cn/). Briefly, chromatographic separations were performed on an ACQUITY UPLC BEH C18 column (2.1 × 100 mm, 1.7 μm) in 0.1% formic acid water (solvent A) and acetonitrile‐isopropanol (9:1, v/v; solvent B) with the following gradient: 0–2 min, 25%–25% B; 2–10 min, 25%–95% B; 10–12 min, 95%–95% B; 12–15 min, 95%–25% B. Flow rate, 0.4 mL/min, temperature, 40°C, injection volume: 10 μL. The negative ion source parameters were optimized as follows: ion spray needle voltage, −4500 V; turbo gas temperature, 550°C; collisional activated dissociation gas, medium level. GS1, GS2, and CUR were set as 50, 50, and 35 psi, respectively. Delustering potential and collision energy values in multiple‐reaction monitoring model were optimized based on our previous studies [40].

### Oxylipin‐rich chow preparation

Considering the instability of oxylipins to oxygen and light conditions, three antioxidants at safe doses, including 5 mg/kg vitamin E (a natural antioxidant of plant and animal oils), 20 mg/kg propyl gallate, and 40 mg/kg tert‐butylhydroquinone (two effective antioxidants against the oxidation of unsaturated fatty acyls) were combined with oxylipins to minimize the influence of oxygen. To minimize the influence of light, the feed preparation was carried out with low‐temperature stirring in light‐sheltered environments. Additionally, the feed was placed in a light‐resistant device throughout the treatment. The feed was renewed every 3 days, and different batches underwent strict quality control by using LC/MS‐based quantitative detection of AROs in chow (the values of relative standard deviation for all deuterated oxylipin standards are shown in Table S3).

### Myocardial infarction reperfusion model and intervention

The experiments and animal handling procedures were conducted in accordance with the NIH Guide for the Care and Use of Laboratory Animals. The animal experiment included ten groups, namely the Sham group, MI/R group, AROC + MI/R group, POC + MI/R group, and six ARO individual + MI/R groups. The number of mice included in each group met the criteria for obtaining a result with statistical significance (*n* ≥ 6): Ten male C57BL/6 mice (aged 8–10 weeks) were included in the Sham group, MI/R group, AROC + MI/R group, and POC + MI/R group. Eight male C57BL/6 mice (aged 8–10 weeks) were included in each individual ARO‐treated group. Before surgical induction of MI/R, AROC, POC, and individual ARO were supplemented through diet for 2 continuous weeks. Then, the mice were anesthetized with pentobarbital (2 mg/kg), a 7–0 surgical silk suture was used to ligate the left anterior descending coronary artery (LAD), and the mice underwent 45 min of LAD‐induced ischemia according to previous study [41]. Considering the lower chow intake and weak physiological status of the mice on the day of surgery and the 3‐day recovery period after thoracotomy, oxylipins were delivered by intraperitoneal injection (10% of dietary additions, twice per day). The dietary oxylipin supplement was subsequently resumed on day four and continued for 2 weeks of reperfusion. The Sham and MI/R mice received vehicle diet‐containing antioxidants or intraperitoneal injection of saline. After treatment, all the mice were deeply anaesthetized via sodium pentobarbital for echocardiographic analysis, and a two‐dimension guided M‐mode echocardiography was performed via a Vevo 2100 Ultrasound System (Visual Sonics Inc.). Then, the plasma and heart tissues were collected. The heart tissue samples were fixed with 4% paraformaldehyde or frozen at −80°C until analysis.

### Cell culture and treatment protocol

RAW 264.7 murine macrophages were acquired from Peking Union Medical College, Cell Bank. The cells were cultured in Dulbecco's modified Eagle's medium supplemented with 10% fetal bovine serum and 1% penicillin‒streptomycin at 37°C in a humidified environment with 5% CO_2_. The cells were seeded into a six‐well plate at a density of 2 × 10^5^ cells/mL and cultured to 70%–80% confluence. Lipopolysaccharide (LPS) was used to induce macrophage polarization, according to our previous work [42]. The cell experiment included three groups, namely the Vehicle group (*n* = 6), the LPS group (*n* = 6), and the LPS + AROC group (*n* = 6). For the AROC treatment group, the cells were rinsed with fresh medium containing AROC (mixtures of 1 μM 14(15)‐EpETE, 2 μM 12,13‐EpOME, 1 μM RvD1, 2.5 μM 6 keto‐PGF1, 2.5 μM 14,15‐EET, and 1 μM LXA4), and stimulated with 1 μg/mL LPS. After incubation for 24 h, the cells were collected for subsequent analysis.

### Histological analysis, biochemistry assays, and western blot

The heart tissues fixed with paraformaldehyde were embedded in paraffin, and then 5 μm paraffin‐embedded heart sections were stained with hematoxylin‐eosin (HE) and Masson's trichrome for cardiomyocyte hypertrophy and cardiac collagen measurements. Heart paraffin sections were also prepared and subjected to double staining with the anti‐cTnT antibody and TUNEL cell death detection kit. DAPI was used for nuclear staining. The frozen heart tissues were removed from the −80°C refrigerator, immediately embedded in Tissue‐Tek OCT compound (Sakura), cut into 10 μm‐thick sections, incubated with dihydroethidium, and mounted with DAPI. Images were obtained by using an Eclipse Ti‐SR fluorescence microscope (Nikon) and Pannoramic MIDI (3DHISTECH Ltd.). Using the Image J software (version 1.49i), all histological datasets were obtained from two random heart tissue regions of three mice in each group. The detailed methods of biochemistry assays and western blot are described in the Supplementary information.

### Untargeted metabolomics and mass spectrometry imaging (MSI) of heart tissues

For untargeted metabolomics, 10 mg of dry heart tissue was mixed with 200 µL of ice‐cold methanol (containing a variety of deuterated chemical standards, as listed in Table S3) followed by homogenization or vortex. Next, 400 µL of MTBE was added, and the mixture was vortexed for another 10 min. Phase separation of the mixture was induced by adding 500 μL of ice‐cold water. Hydrophilic and lipophilic supernatants were obtained and dried under vacuum. The dried residue was redissolved in 100 μL of methanol–water (1:1, v/v) for further analysis. Untargeted metabolomics analysis of heart tissues was performed on a UPLC‐SYNAPT Xevo‐G2 XS Q‐TOFMS system (Waters Corporation). Chromatographic separation was achieved through an ACQUITY UPLC BEH C18 column (100 × 2.1 mm, 1.7 µm, Waters Corp.). The LC/MS conditions were optimized based on our previous work [43], and the detailed conditions of chromatographic separation and mass spectrometry are described in the Supplementary Information. The spatial distribution of ceramides in heart tissue was determined via a Xevo‐G2 XS Q‐TOF mass spectrometer (Waters) equipped with a two‐dimensional DESI‐MSI platform (Indianapolis). The protocols for the preparation of frozen specimens and MSI analysis were optimized based on our previous work [44]. The detailed method is described in the Supplementary Information.

### Targeted analysis of plasma ceramides

Targeted profiles of ceramides (Cer, d18:1/16:0; d18:1/24:1; d18:1/16:0) in plasma samples were performed by MetWare (http://www.metware.cn/) following standard protocols. Briefly, 400 μL of cold methanol/acetonitrile (1:1, v/v) extraction solvent was added to remove the protein, extract the ceramides, and then adequately vortexed. For absolute quantification of ceramides, stock solutions of stable‐isotope ceramides [Cer‐d_*7*_ (d18:1/16:0), Cer‐d_*7*_ (d18:1/16:0), and Cer‐d_*7*_ (d18:1‐d7/24:1(15Z)] were added to the extraction solvent simultaneously. The mixture was subsequently centrifuged at 14,000 rpm for 20 min at 4°C. The supernatant was collected and dried in a vacuum centrifuge and re‐dissolved in 100 μL of acetonitrile/water (1:1, v/v) for further analysis. Analyses were performed using an UHPLC (1290 Infinity LC, Agilent Technologies) coupled to a QTRAP MS (6500+, Sciex). The analytes were separated on C18 columns (Waters UPLC BEH C18‐100 × 2.1 mm, 1.7 μm). The flow rate of the mobile phase remained constant at 0.4 mL/min. MS was performed in positive switch mode. The ESI positive source conditions were as follows: source temperature: 580°C; ion source Gas1: 45 psi; ion source gas2: 60 psi; curtain gas: 35 psi; ion spray voltage: +4500 V. The multiple‐reaction monitoring model was used for mass spectrometry quantitative data acquisition.

### Metabolic pathway, functional enrichment, and correlation analysis

MetaboAnalyst (http://www.metaboanalyst.ca/) software was used to investigate the most relevant pathways of the ARO‐altered metabolites. The relationship networks among ARO, ARO‐altered metabolite features, associated biological functions, and involved diseases were generated by using the connect analysis and pathway explorer modules in the Ingenuity Pathway Analysis software (IPA, QIAGEN Inc., German). The correlation between ARO metabolites and biochemical factors was assessed by Spearman's rank coefficient plot using the bioinformatics platform (http://www.bioinformatics.com.cn); the significant thresholds were coefficients ≥0.5 and FDR‐adjusted *p* values < 0.05.

### Statistical analysis

For clinical variables, continuous data are presented as the means and standard deviations (means ± SDs), and the non‐normally distributed data are expressed as medians and interquartile range [IQR]. The unpaired two‐tailed Student's *t‐*test and Mann–Whitney *U* test were used for two‐group comparisons of normally distributed data and non‐normally distributed data, respectively. Categorical variables are summarized by frequency (*N*) and percentages (%) and were compared using the chi‐square test. For the animal and cellular variables, all statistical comparisons were independently evaluated using Student's *t*‐test. All the comparisons were performed by using GraphPad Prism 8.0 (GraphPad Software Inc.), and a *p*‐value < 0.05 was considered significant. For animal study, the number of mice included in the biochemical and metabolomic analyses met the statistical criteria (*n* ≥ 6 in each group), and all the included mice were randomly selected by a nonexperimental operator. Due to the amounts of the plasma or heart tissue samples might not be sufficiently available for all biochemical analysis; therefore, the included sample sizes in different assays might not be consistent.

Multivariate statistical analysis of the data matrix of PUFAs and their oxygenated oxylipins was performed by SIMCA‐P software (version 14.0, Umetric). Unsupervised PCA was first employed to assess the quality, homogeneity, and outliers of the data set and explore the group separations. The variable influence in the projection (VIP ≥ 1.5) plot derived from supervised PLS‐DA was subsequently performed to identify the differentially expressed variables. The selected variables were subsequently confirmed by two‐tailed Student's *t*‐test or Mann–Whitney *U* test (FDR‐adjusted *p* < 0.05). Beta‐coefficient analysis for investigating the association between the detected oxylipins and clinical variables was performed via SPSS Statistics software (version 26, IBM Corp).

The RF‐based machine learning algorithm was used to estimate the associations between the detected oxylipins and recurrent MACE and identify important risk markers. The RF algorithm was performed via R software (version 4.3.1) and MetaboAnalyst software (version 4.3.1), as previously described [45]. Briefly, the ROSE method was applied to reduce the data imbalance rate between non‐recurrent MACE and recurrent MACE. The *ntree* value and mtry value were set at 500 and 5, and the other hyperparameters were set at default settings. The importance of each oxylipin was evaluated by using the plot of mean decrease accuracy. Monte Carlo cross‐validation was employed to generate multivariate RF models and screen the optimal risk marker panel in the discovery cohort. In each Monte Carlo cross‐validation, 70% of the samples were used to evaluate the performance of different RF models with different numbers of top oxylipin markers. The remaining 30% of the samples were subsequently used to test the performances of the established RF models. The performance and reliability of the RF‐based risk model were assessed by multivariate RF model‐based ROC curves and the number of misclassifications in the posterior classification probability plot with 200 cross‐validation (AUC‐ROC ≥ 0.8 and proportion of incorrect classifications <20% were considered significant). To assess the performances of different RF‐based risk models, the DeLong test of the different RF model‐derived AUC‐ROC values was performed by Med Calc software (version 20.1), and a *p*‐value < 0.05 was considered significant.

## AUTHOR CONTRIBUTIONS

**Zhiyong Du**: Conceptualization; investigation; funding acquisition; writing—review and editing; writing—original draft; validation; methodology; project administration; resources. **Yingyuan Lu**: Conceptualization; methodology; investigation; writing—review and editing; writing—original draft; funding acquisition. **Ying Ma**: Investigation; writing—original draft; writing—review and editing; funding acquisition. **Yunxiao Yang**: Investigation; writing—original draft. **Wei Luo**: Investigation; writing—original draft. **Sheng Liu**: Investigation; writing—original draft. **Ming Zhang**: Investigation; writing—original draft. **Yong Wang**: Investigation; writing—original draft; validation; visualization. **Lei Li**: Investigation; validation; methodology; writing—original draft; conceptualization. **Chun Li**: Investigation; conceptualization; methodology; validation; visualization; writing—original draft; writing—review and editing; project administration; funding acquisition. **Wei Wang**: Conceptualization; project administration; funding acquisition; writing—original draft; writing—review and editing; investigation. **Hai Gao**: Conceptualization; investigation; methodology; validation; project administration; writing—review and editing; writing—original draft; funding acquisition.

## CONFLICT OF INTEREST STATEMENT

The authors declare no conflicts of interest.

## ETHICS STATEMENT

The ethics applications (Nos. 077‐02, 2020025X, KS2023049, and BUCM‐2023050501‐2072) were approved by the Ethics Committee of Beijing Anzhen Hospital of the Capital University of Medical Sciences, Peking University Third Hospital, and Beijing University of Chinese Medicine.

## Supporting information


**Figure S1.** Beta‐coefficient plots depicting the associations of the detected oxylipins with age and sex.
**Figure S2.** Beta‐coefficient plots depicting the associations of the detected oxylipins with previous cardiovascular events and aspirin use.
**Figure S3.** Beta‐coefficient plots depicting the associations of the detected oxylipins with the plasma levels of hypersensitive C‐reactive protein and brain natriuretic peptide.
**Figure S4.** Beta‐coefficient plots depicting the associations of the detected oxylipins with troponin T/I and body mass index.
**Figure S5.** Beta‐coefficient plots depicting the associations of the detected oxylipins with hypertension and type 2 diabetes mellitus.
**Figure S6.** Multivariate random forest algorithm‐based oxylipin marker selection.
**Figure S7.** Receiver operating curves generated from Monte Carlo cross‐validation‐based multivariate random forest models using different numbers of top anti‐inflammatory/pro‐resolving oxylipin markers.
**Figure S8.** Time‐dependent dynamic assessments of the stability of oxylipins in the diets under dark and light conditions.
**Figure S9.** Optimal dose selection and quality assessment of the oxylipins in the diets.
**Figure S10.** Evaluation of the effects of six individual ARO treatment on myocardial ischemia‒reperfusion model mice.
**Figure S11.** Effects of the combined treatment of anti‐inflammatory/pro‐resolving oxylipins on the myocardial metabolome and proinflammatory marker levels in myocardial ischemia‒reperfusion model mice.
**Figure S12.** Optimization of oxylipins extraction methods for plasma sample.

**Table S1.** Demographic and clinical baseline characteristics of patients with STEMI in the discovery and external validation cohorts.
**Table S2.** The full list of oxylipin and polyunsaturated fatty acid standards.
**Table S3.** Relative standard deviation values for all deuterated standard substances in the quality control samples.
**Table S4.** Demographic and clinical characteristics of the matched patients with ST‐segment elevation myocardial infarction and healthy individuals.

## Data Availability

The data that support the findings of this study are available from the corresponding author upon reasonable request. The normalized oxylipin profiling datasets have been deposited in the OMIX, China National Center for Bioinformation/Beijing Institute of Genomics, Chinese Academy of Sciences (accession number: OMIX008181, https://ngdc.cncb.ac.cn/omix/preview/c77udIaG; accession number: OMIX008182, https://ngdc.cncb.ac.cn/omix/preview/zvJIBpc2). The data and scripts used are saved in Gitee (https://gitee.com/imeta-2024-stemi/oxylipins-STEMI). Supplementary materials (methods, figures, tables, graphical abstract, slides, videos, Chinese translated version, and update materials) may be found in the online DOI or iMeta Science http://www.imeta.science/.
