# Supplementary material for: The prognostic and therapeutic significance of polyunsaturated fatty acid‐derived oxylipins in ST‐segment elevation myocardial infarction
Source: Imeta. 2025 Jan 9;4(1):e266. doi: 10.1002/imt2.266 (PMC11865345; doi:10.1002/imt2.266)
Supplement: Supplementary file 1 — Figure S1. Beta‐coefficient plots depicting the associations of the detected oxylipins with age and sex. Figure S2. Beta‐coefficient plots depicting the associations of the detected oxylipins with previous cardiovascular events and aspirin use. Figure S3. Beta‐coefficient plots depicting the associations of the detected oxylipins with the plasma levels of hypersensitive C‐reactive protein and brain natriuretic peptide. Figure S4. Beta‐coefficient plots depicting the associations of the detected oxylipins with troponin T/I and body mass index. Figure S5. Beta‐coefficient plots depicting the associations of the detected oxylipins with hypertension and type 2 diabetes mellitus. Figure S6. Multivariate random forest algorithm‐based oxylipin marker selection. Figure S7. Receiver operating curves generated from Monte Carlo cross‐validation‐based multivariate random forest models using different numbers of top anti‐inflammatory/pro‐resolving oxylipin markers. Figure S8. Time‐dependent dynamic assessments of the stability of oxylipins in the diets under dark and light conditions. Figure S9. Optimal dose selection and quality assessment of the oxylipins in the diets. Figure S10. Evaluation of the effects of six individual ARO treatment on myocardial ischemia‒reperfusion model mice. Figure S11. Effects of the combined treatment of anti‐inflammatory/pro‐resolving oxylipins on the myocardial metabolome and proinflammatory marker levels in myocardial ischemia‒reperfusion model mice. Figure S12. Optimization of oxylipins extraction methods for plasma sample. [file IMT2-4-e266-s001.docx]

**Supporting information to**

# The prognostic and therapeutic significance of polyunsaturated fatty acid-derived oxylipins in ST-segment elevation myocardial infarction

**Running title:** Prognostic and therapeutic values of bioactive oxylipins in STEMI

Zhiyong Du ^1#^, Yingyuan Lu ^3#^, Ying Ma ^2#^, Yunxiao Yang ^1,4^, Wei Luo ^1,4^, Sheng Liu ^1,4^, Ming Zhang ^1,4^, Yong Wang ^5^, Lei Li ^6^**^*^**, Chun Li ^7,8^**^*^**, Wei Wang ^7^**^*^**, Hai Gao ^1,4^**^*^**

^1^ Key Laboratory of Remodeling-Related Cardiovascular Diseases, Ministry of Education, National Clinical Research Center for Cardiovascular Diseases, Beijing Institute of Heart Lung and Blood Vessel Disease, Beijing Anzhen Hospital, Capital Medical University, Beijing 100029, China

^2^ The State Key Laboratory for Quality Ensurance and Sustainable Use of Dao-di Herbs, National Resource Center for Chinese Materia Medica, China, Academy of Chinese Medical Sciences, Beijing 100700, China

^3^ School of Pharmaceutical Sciences, State Key Laboratory of Natural and Biomimetic Drugs, Peking University, Beijing 100191, China

^4^ Department of Cardiology, Beijing Anzhen Hospital, Capital Medical University, Beijing 100029, China

^5^ Dongzhimen Hospital of Beijing University of Chinese Medicine, Beijing 100700, China

^6^ Department of Cardiology, Peking University Third Hospital, Beijing 100191, China

^7^ State Key Laboratory of Traditional Chinese Medicine Syndrome, Guangdong Provincial Key Laboratory of Syndrome and Formula, Guangzhou 510700, China

^8^ Modern Research Center for Traditional Chinese Medicine, Beijing University of Chinese Medicine, Beijing 100029, China

^#^ These authors contributed equally: Zhiyong Du, Yingyuan Lu, Ying Ma

**^*^**Correspondence: gaohai1221@mail.ccmu.edu.cn (Hai Gao), wangwei26960@126.com (Wei Wang), lichun19850204@163.com (Chun Li), dr_lilei@bjmu.edu.cn (Lei Li).

# Supplementary Method

## Optimization of plasma polyunsaturated fatty acid and oxylipin extraction

Solid-phase extraction (SPE) method: 50 μL of plasma sample was added 25% (v/v) acetonitrile to 1 mL, vortexed for 2 min, and applied to Waters Oasis MAX 3 cc Vac Cartridge SPE cartridges that had been preconditioned with 1 mL acetonitrile followed with 1 mL of 25% (v/v) acetonitrile. The cartridges were then washed with 1 mL of 25% (v/v) acetonitrile, and 1 mL of acetonitrile in succession. Finally, the oxylipins were eluted with 1 mL of 1% formic acid in acetonitrile.

Protein precipitation (PPT) combined with liquid‒liquid extraction (LLE) method 1: 200 μL of ice-cold solvent consisting of methanol-deionized water (1:1; v/v) with 1% formic acid was added to 50 μL of plasma sample, and the mixture was subsequently vortexed for 2 min and centrifuged at 15000 rpm for 15 min at 4 °C. The upper supernatants were collected as upper phase I. Subsequently, 200 μL of ice-cold MTBE/methanol (7:3, v/v) with 1% formic acid was added to the residual precipitate, and the upper supernatants were collected as upper phase II, which combined with upper phase I.

PPT combined with LLE method 2: 50 μL of plasma sample was added 200 μL of ice-cold methanol with 1% formic acid, and the mixture was subsequently vortexed for 2 min and centrifuged at 15000 rpm for 15 min at 4 °C. The upper supernatants were collected as upper phase I. Subsequently, 200 μL of ice-cold MTBE/methanol (7:3, v/v) with 1% formic acid was added to the residual precipitate, and the upper supernatants were collected as upper phase II, which combined with upper phase I.

## Chromatographic separation and mass spectrometry conditions of the untargeted metabolomics of heart tissues

The column was maintained at 40 °C, and the flow rate was 0.4 mL/min, and a total of 5 μL aliquot of each sample was injected. The mobile phase consisted of a linear gradient system of (Solvent A) water and (Solvent B) 0.1% ammonium acetate in acetonitrile. The source temperature was set at 110 °C. The capillary voltage was 3.0 kV for positive ion mode (ESI^+^) and 2.5 kV for negative ion model (ESI^−^), respectively. The desolvation gas temperature and desolvation gas flow were 450 °C and 700 L/h, respectively. All analyses were acquired using a LockSpray interface. LE was used as the reference compound with m/z 556.2771 for ESI^+^ and m/z 554.2615 for ESI^−^. The collision energy parameters ranged from 5 eV to 60 eV. Metabolite identification was performed by using the in-house MS/MS databases of authentic standards and the offline Progenesis QI databases, including MetaScope, HMDB, METLIN, and LIPIDMAPS.

## Anti-inflammatory/pro-resolving oxylipin extraction and quantification of the heart tissues and plasma samples from mice

The frozen heart tissues of the mice were thawed at 4 °C, and 10 mg of each sample was precipitated by adding 200 μL of ice-cold methanol with 1% formic acid containing the deuterated anti-inflammatory/pro-resolving oxylipin (ARO) standards (the detailed concentrations are shown in Table S3) followed by homogenization or vertex. The mixture was subsequently vortexed for 2 min and centrifuged at 15000 rpm for 15 min at 4 °C, and the upper supernatants were collected as upper phase I. Subsequently, 200 μL of ice-cold MTBE/methanol (7:3, v/v) with 1% formic acid was added to the residual precipitate. After vortex-blending for 2 min, the mixture was centrifuged at 15000 rpm for 15 min at 4 °C. The upper supernatants were collected as upper phase II and combined with upper phase I. Prior to analysis, the eluent was dried under vacuum and redissolved in 100 μL of methanol-acetonitrile (1:1, v/v) for UPLC/MS/MS analysis. For the plasma samples from the mice, the ARO extraction methods were consistent with the oxylipin extraction methods used for human plasma samples. The levels of ARO were detected by AB Sciex QTRAP LC‒MS/MS platform. The chromatographic separations were performed on an ACQUITY UPLC BEH C18 column (2.1 × 100 mm, 1.7 μm) in 0.1% formic acid water (Solvent A) and acetonitrile-isopropanol (9:1, v/v; Solvent B) with the following gradient: 0–2 min, 25%–25% B; 2–10 min, 25%–95% B; 10–12 min, 95%–95% B; 12–15 min, 95%–25% B. Flow rate: 0.4 mL/min; temperature: 40 °C; injection volume: 10 μL; ion spray needle voltage, − 4500 V; turbo gas temperature: 550 °C. GS1, GS2, and CUR were set as 50 psi, 50 psi, and 35 psi, respectively.

## Mass spectrometry imaging (MSI) of ceramide in heart tissues by DESI-MSI platform

Two frozen heart tissues were randomly selected from each group. Ten μm frozen specimens of cross-sectional heart tissue were affixed to glass slides for MSI-DESI‒MSI analysis. The DESI-Q-TOFMS/MS settings were meticulously adjusted to enhance signal clarity, with adjustments made to spatial resolution (100 μm), spray solvent composition (methanol: water, 9:1, v/v) augmented with 0.1 mmol /L NH_4_Cl and 0.1 mmol/L LE, solvent flow rate and raster velocity were set at 1.5 μL/min and 400 μm/s, respectively. The other main parameters were set as follows: sprayer orientation angle (70 °degrees), distances from sprayer to inlet (5 mm), sprayer to specimen (1.5 mm), source heat (130 °C), negative capillary voltages (4.0 kV), cone voltage (85 V). The identification of ceramides in DESI-MSI was mainly based on the primary parent ions and selective secondary daughter ions of the chemical standards of Ceramide (Cer d18:1/16:0), Cer(d18:1/16:0), and Cer(d18:1/24:1(15Z)) in Q-TOF mass spectrometer. The spatial images were processed using high-definition imaging software (version 2.1 Waters, Manchester, U.K.).

## Biochemistry assays and western blot

The plasma lactate dehydrogenase (LDH) and creatine kinase-MB (CK)-MB concentrations were measured via an automated Hitachi 7080 biochemical analyzer (Hitachi High-Technologies Corporation, Japan). Tumor necrosis factor-α (TNF-α), interleukin-1β (IL-1β) and IL-6 levels were evaluated via specific enzyme-linked immunosorbent assay (ELISA) kits following the manufacturer’s protocols. The protein levels in the heart tissues and macrophages were measured via western blotting method. Briefly, proteins were extracted according to the manufacturer’s instructions. The protein concentration of each sample was measured with a bicinchoninic acid (BCA) kit (Beijing Pulilai Gene Technology Co., Ltd., Beijing, China). Equal amounts of protein were loaded on 10% SDS‒PAGE gels and transferred onto PVDF membranes. The membranes were first incubated overnight with primary antibody at 4 °C and secondary antibody for 1.5 h at room temperature and then treated with ECL (GE Healthcare, United States) for 1 min at room temperature. The bands in the membrane were visualized and analyzed via Image Lab software. The final reported data for each target protein were normalized to the data for glyceraldehyde-3-phosphate dehydrogenase (GAPDH).


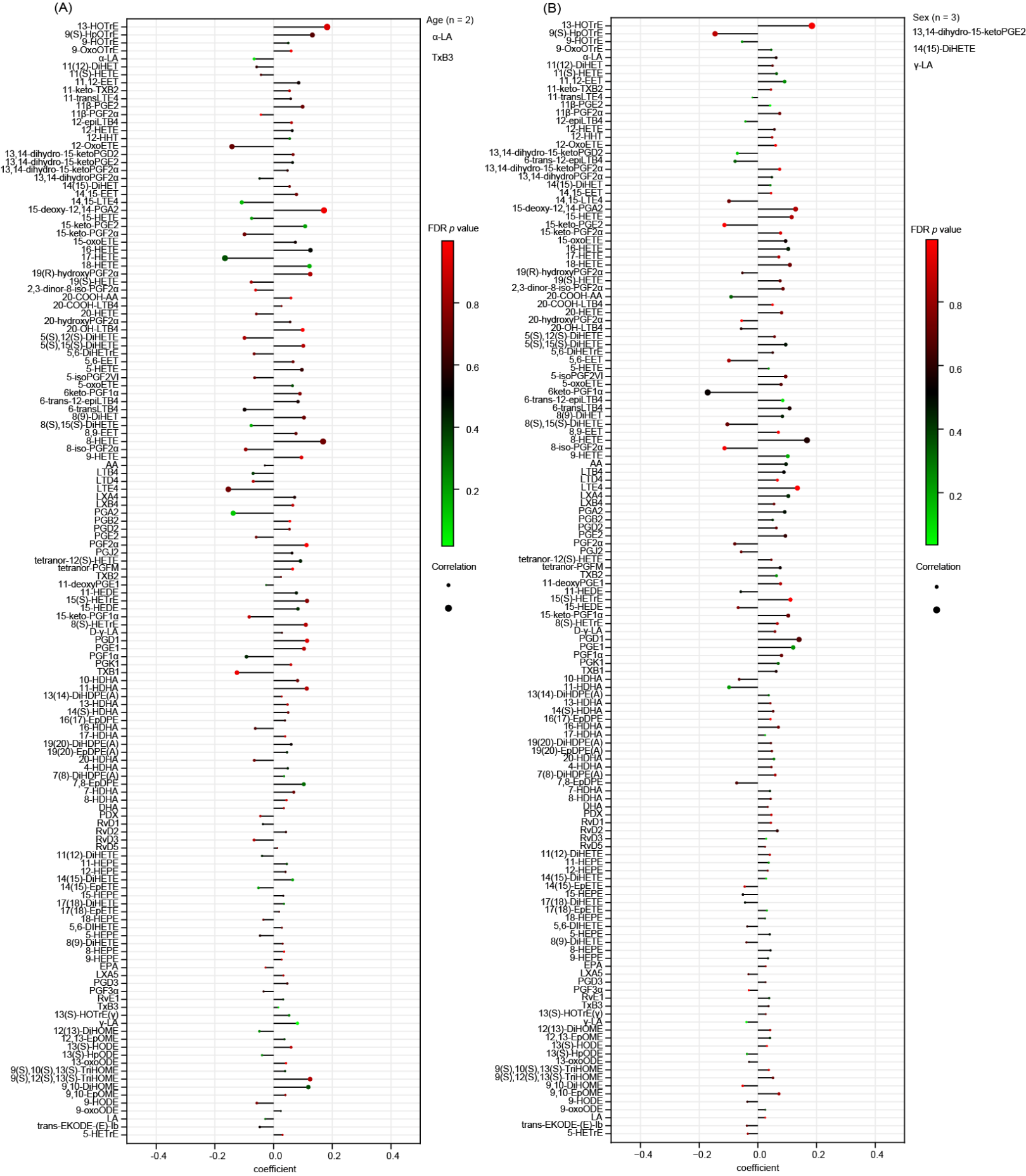


# **Figure S1 Beta-coefficient** **plots depicting the associations of the detected oxylipins with age and sex.** (A) Ages. (B) Sex. Positive or negative beta-coefficient values indicate a positive or negative correlation. FDR-adjusted *p* < 0.05 was considered the significance threshold, and all variables with significant associations are labeled in the legends. Abbreviations: ARA, arachidonic acid; ALA, alpha-linolenic acid; DHA, docosahexaenoic acid; DGLA, dihomo-γ-linolenic acid; DiHDPE, dihydroxy-docosapentaenoic acid; DiHET, dihydroxy-eicosatrienoic acid; DiHETE, dihydroxy-eicosatetraenoic acid; DiHOME, dihydroxy-octadecenoic acid; EET, epoxy-eicosatrienoic acid; EpDPE, epoxy-docosapentaenoic acid; EPA, eicosapentaenoic acid; EpETE, epoxy-eicosatetraenoic acid; EpOME, epoxy-octadecenoic acid; GLA, gamma linolenic acid; HDHA, hydroxy-docosahexaenoic acid; HEDE, hydroxy-eicosadienoic acid; HEPE, hydroxy-eicosapentaenoic acid; HETE, hydroxy-eicosatetraenoic acid; HETrE, hydroxy-eicosatrienoic acid; HODE, hydroxy-octadecadienoic acid; HOTrE, hydroxy-octadecatrienoic acid; HpODE, hydroxy-octadecatrienoic acid; LA, linoleic acid; oxo-EET, oxo-eicosatetraenoic acid; MACE, major adverse cardiovascular events; oxoODE, oxo-octadecadienoic acid.


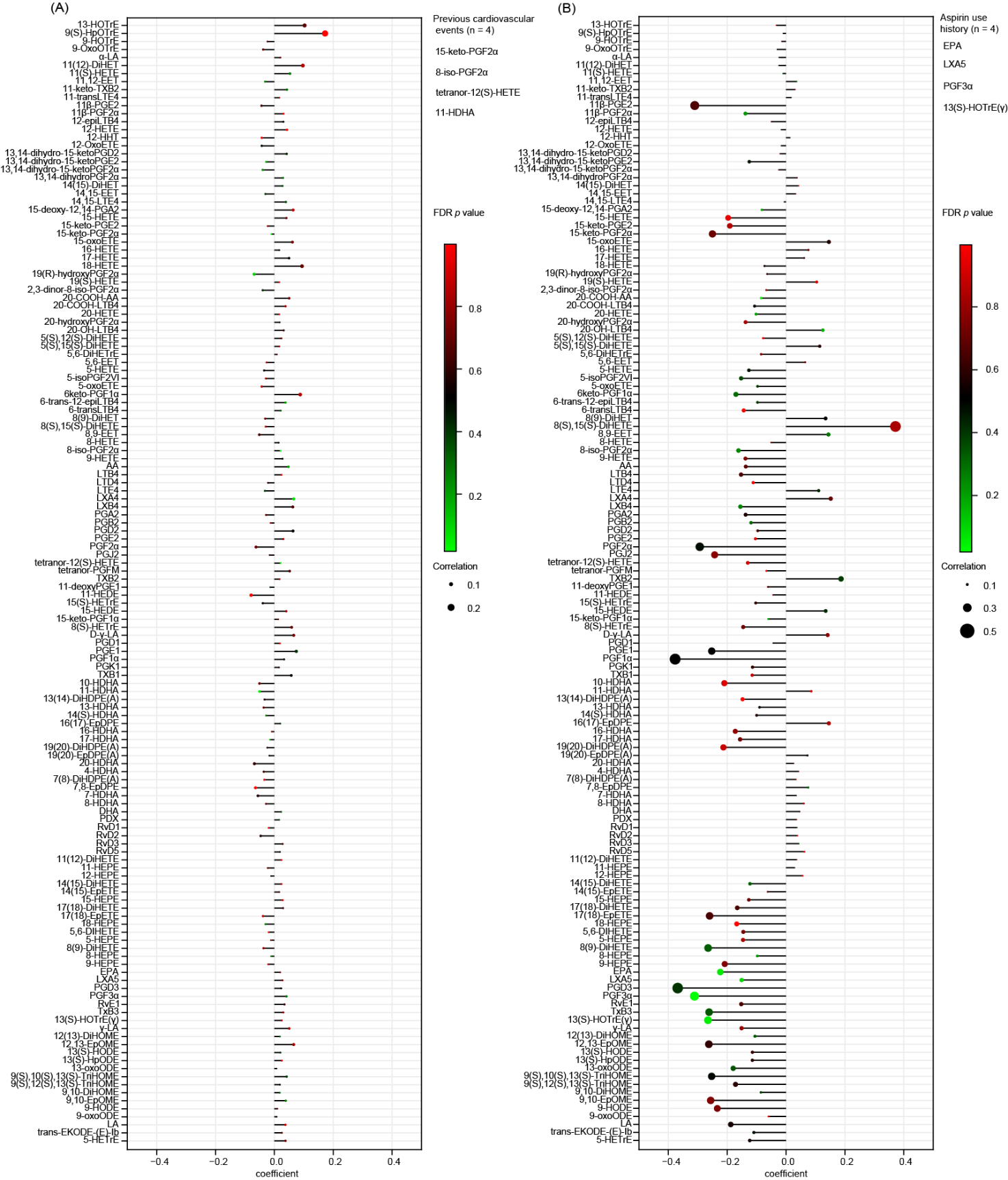


# **Figure S2 Beta-coefficient plots depicting the associations of the detected oxylipins with previous cardiovascular events and** **aspirin use.** (A) Previous cardiovascular events. (B) Aspirin use. Positive or negative beta-coefficient values indicate a positive or negative correlation. FDR-adjusted *p* < 0.05 was considered as significant threshold, and all variables with significant associations are labeled in the legends. Previous cardiovascular events included previous myocardial infarction or primary percutaneous coronary intervention.


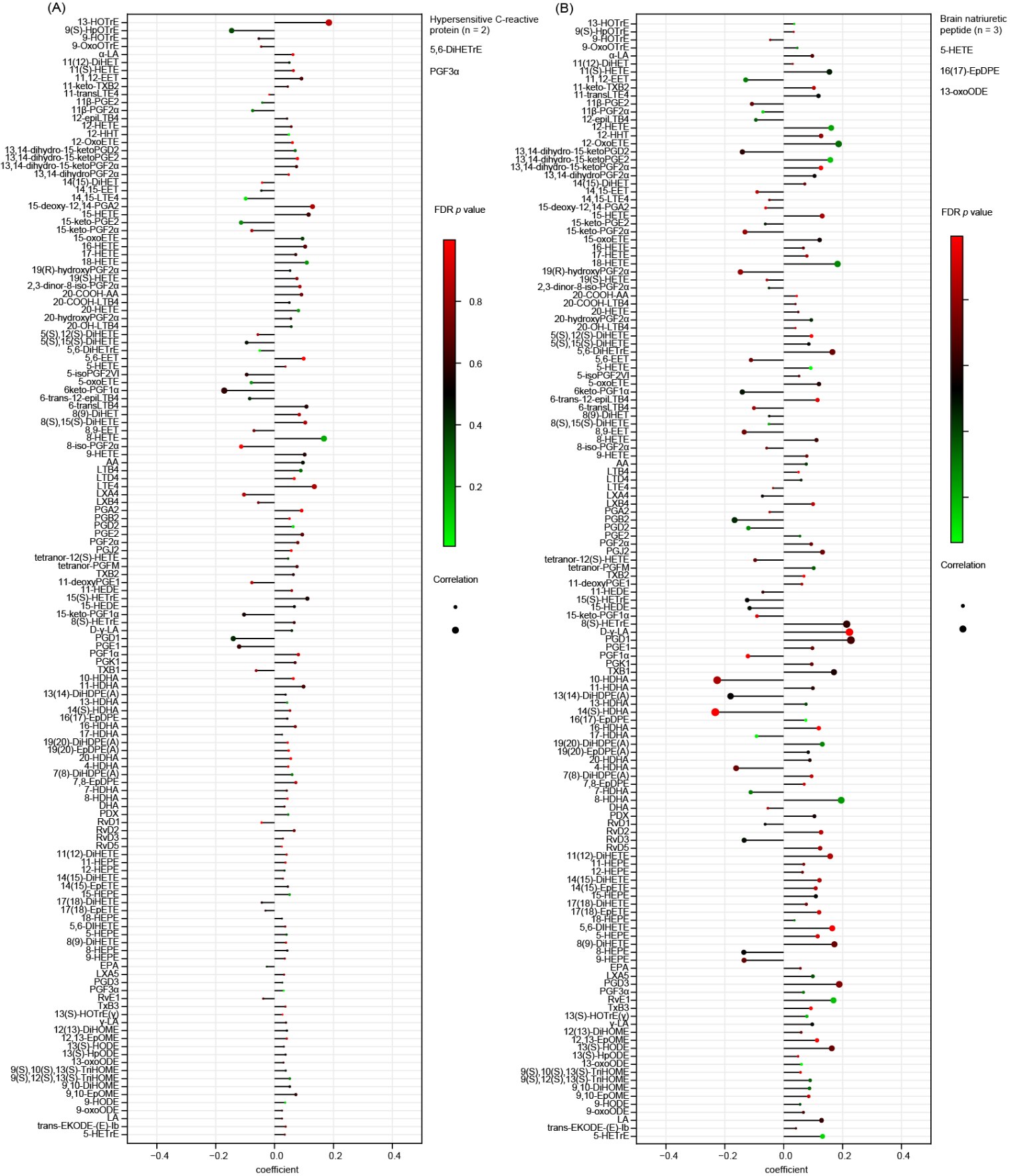


# **Figure S3 Beta-coefficient plots depicting the associations of the detected oxylipins with the plasma levels of hypersensitive C-reactive protein and brain natriuretic peptide.** (A) Hypersensitive C-reactive protein. (B) Brain natriuretic peptide. Positive or negative beta-coefficient values indicate a positive or negative correlation. FDR-adjusted *p* < 0.05 was considered as significant threshold, and all variables with significant associations are labeled in the legends.


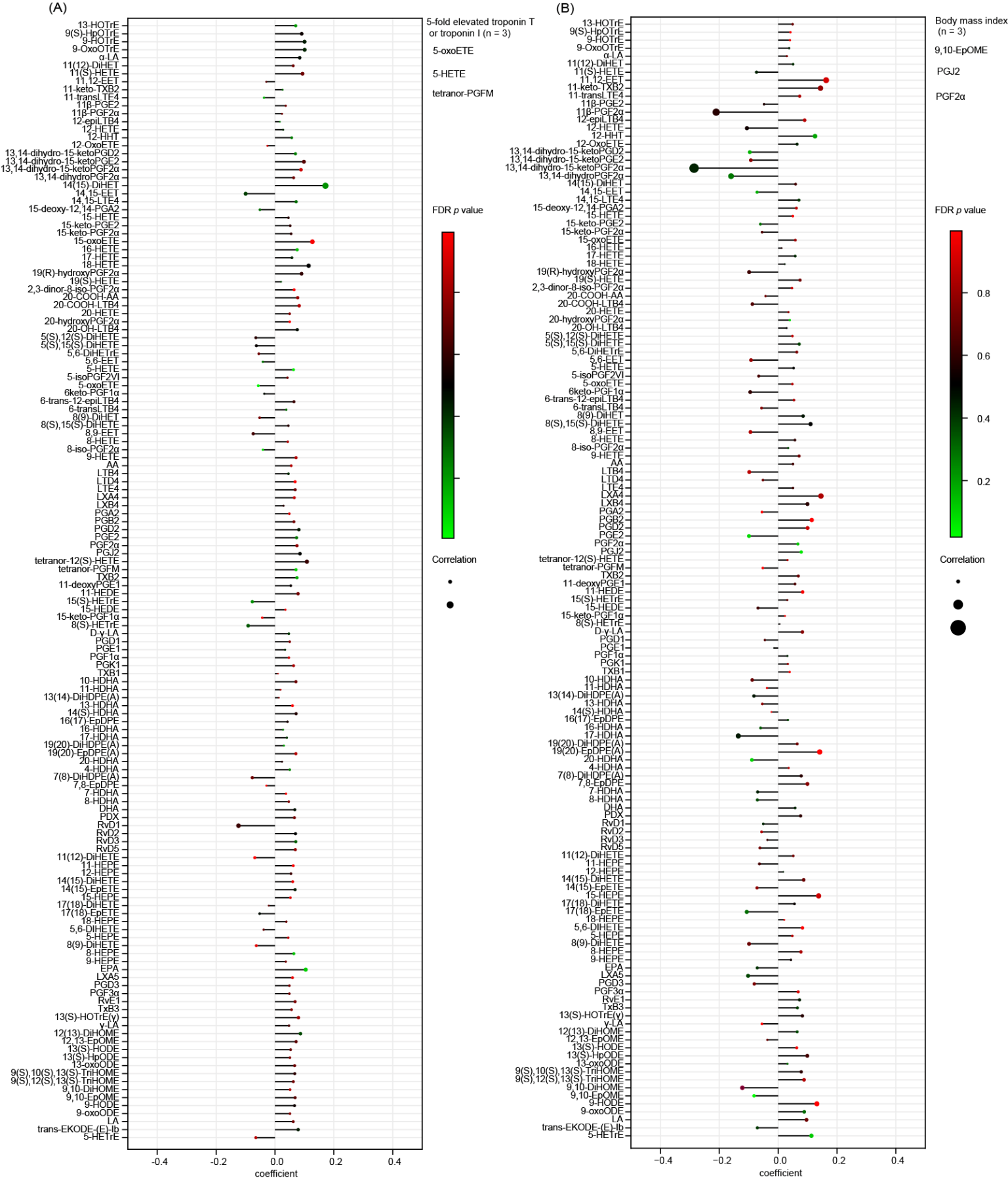


# **Figure S4 Beta-coefficient plots depicting the associations of the detected oxylipins with troponin T/I and** **body mass index.** (A) 5-fold elevated troponin T or troponin I. (B) Body mass index. Positive or negative beta-coefficient values indicate a positive or negative correlation. FDR-adjusted *p* < 0.05 was considered as significant threshold, and all variables with significant associations are labeled in the legends.


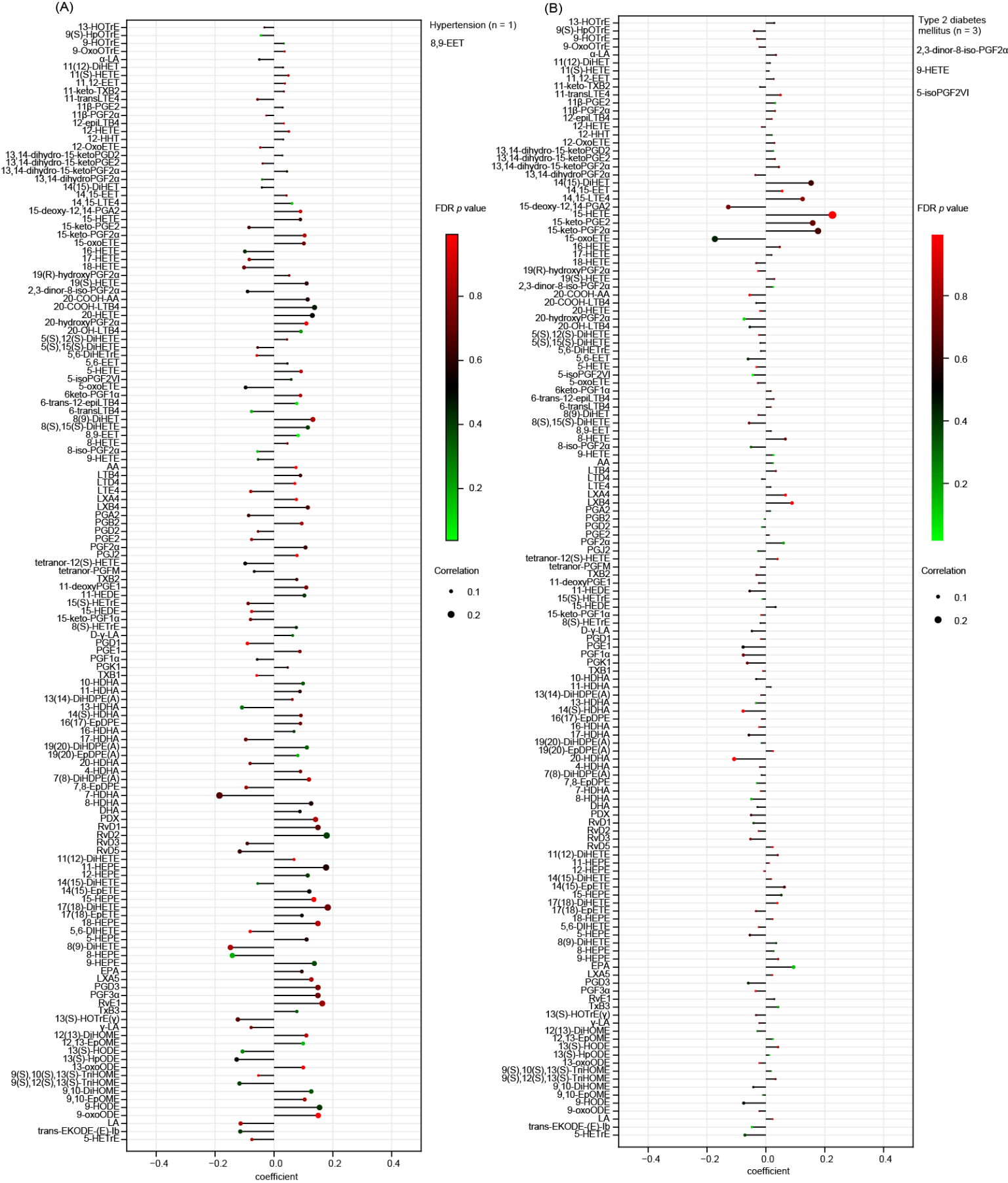


# **Figure S5 Beta-coefficient plots depicting the associations of the detected oxylipins with hypertension and type 2 diabetes mellitus.** (A) Hypertension. (B) Type 2 diabetes mellitus. Positive or negative beta-coefficient values indicate a positive or negative correlation. FDR-adjusted *p* < 0.05 was considered as significant threshold, and all variables with significant associations are labeled in the legends.


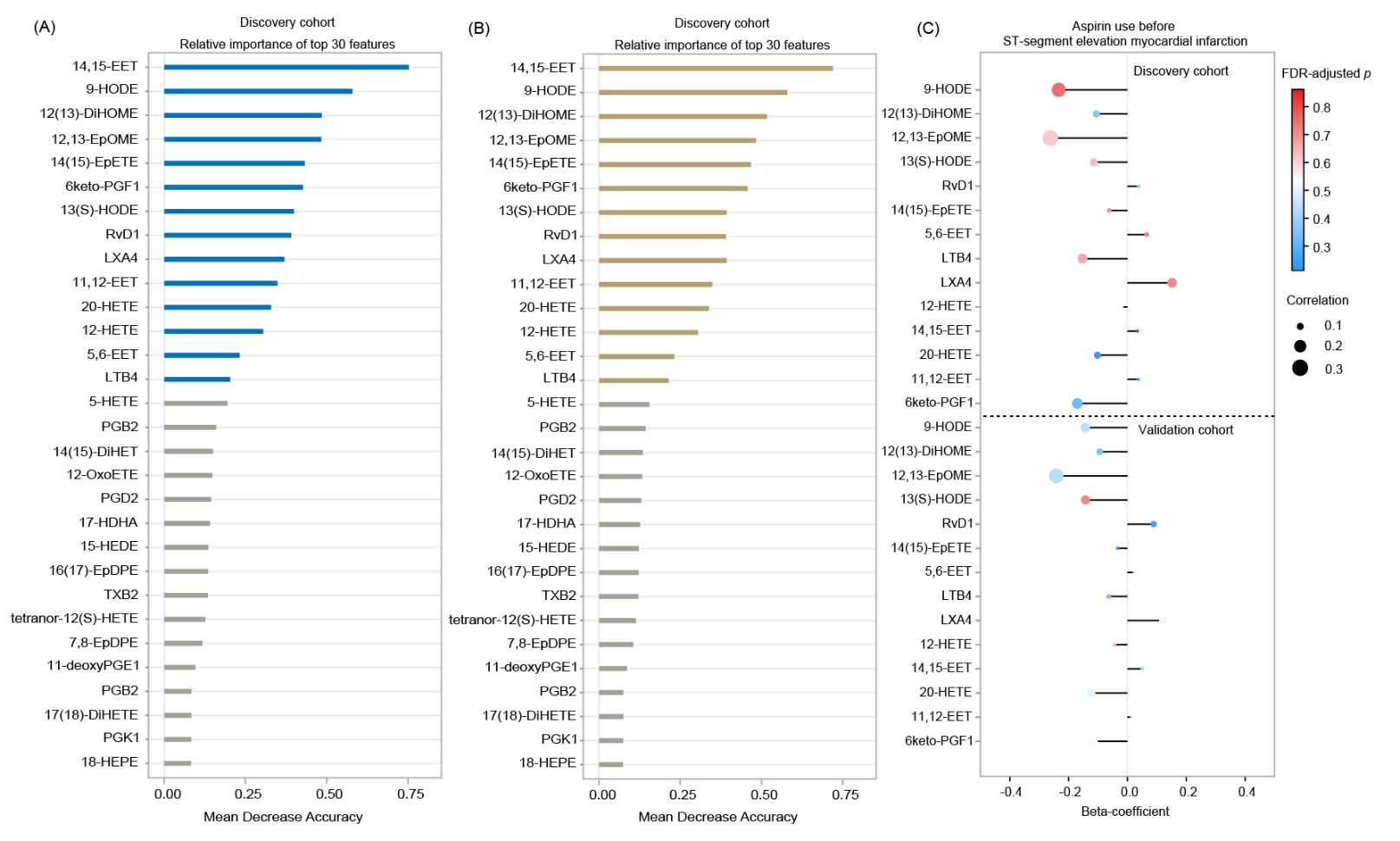


# Figure S6 Multivariate random forest algorithm-based oxylipin marker selection. (A) Unadjusted multivariate random forest-based selection plot of the top-thirty markers in the discovery cohort. (B) Age- and sex-adjusted multivariate random forest marker selection plot for the discovery cohort. (C) Beta-coefficient plot depicting non-statistical associations between the top 14 oxylipin markers and aspirin use (FDR-adjusted *p* > 0.05). Abbreviations: 14,15-EET, 14(15)-epoxy-eicosatrienoic acid; 9-HODE, 9-hoctadecadienoic acid; 12(13)-DIHOME, 12(S),13(S)-dihydroxyoctadecadienoic acid; 12,13-EpOME, 12(13) epoxy-octadecenoic acid; TXB1, thromboxane B1; 17-HDHA, 17-hydroxy-docosahexaenoic acid; 11-keto-TXB2, 11-keto-thromboxane B2; 6keto-PGF1, 6 keto-prostaglandin F1; RvD1, resolvin D1; LXA4, lipoxin A4; 12-HETE, 20-hydroxyeicosatetraenoic acid; 20-HETE, 12-hydroxyeicosatetraenoic acid; LTB4, leukotriene B4.


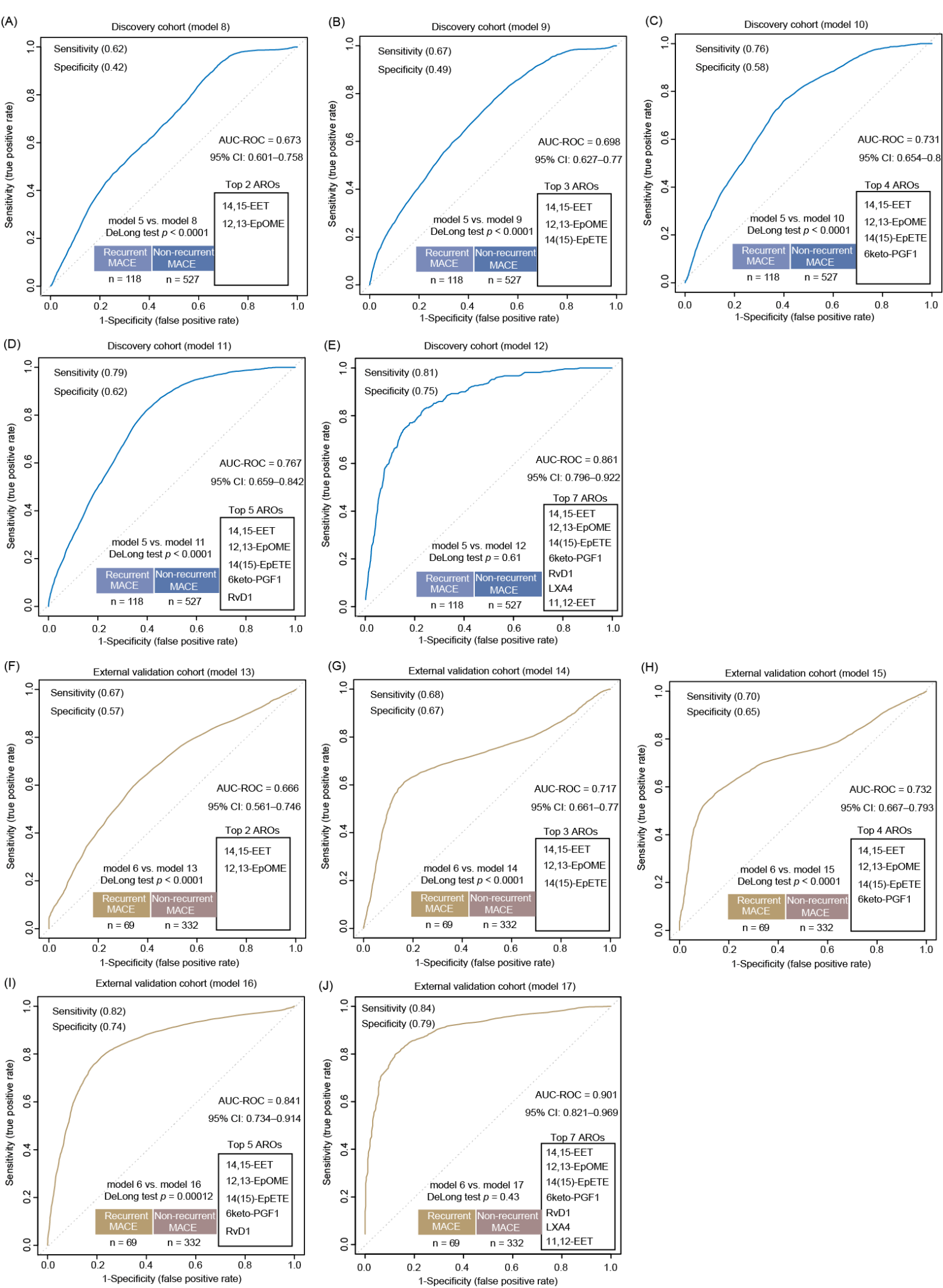


# Figure S7 Receiver operating curves generated from Monte Carlo cross-validation-based multivariate random forest models using different numbers of top anti-inflammatory/pro-resolving oxylipin markers. (A-E), Receiver operating curve (ROC) based on the top-two anti-inflammatory/pro-resolving oxylipin (ARO) (model 8), top-three ARO (model 9), top-four ARO (model 10), top-five ARO (model 11), and top-seven ARO (model 12) in the discovery cohort. (F-J) ROC based on the top-two ARO (model 13), top-three ARO (model 14), top-four ARO (model 15), top-five ARO (model 16), and top-seven ARO (model 17) in the external validation cohort. A DeLong test *p* < 0.05 indicates a significant difference in the area under ROC curve (ROC-AUC) values of different RF risk models compared with the ROC-AUC values of model 5 (top-six AROs: 14,15-EET, 12,13-EpOME, 14(15)-EpETE, 6keto-PGF1, RvD1, and LXA4) in the discovery cohort or model 6 (top-six AROs) in the external validation cohort.


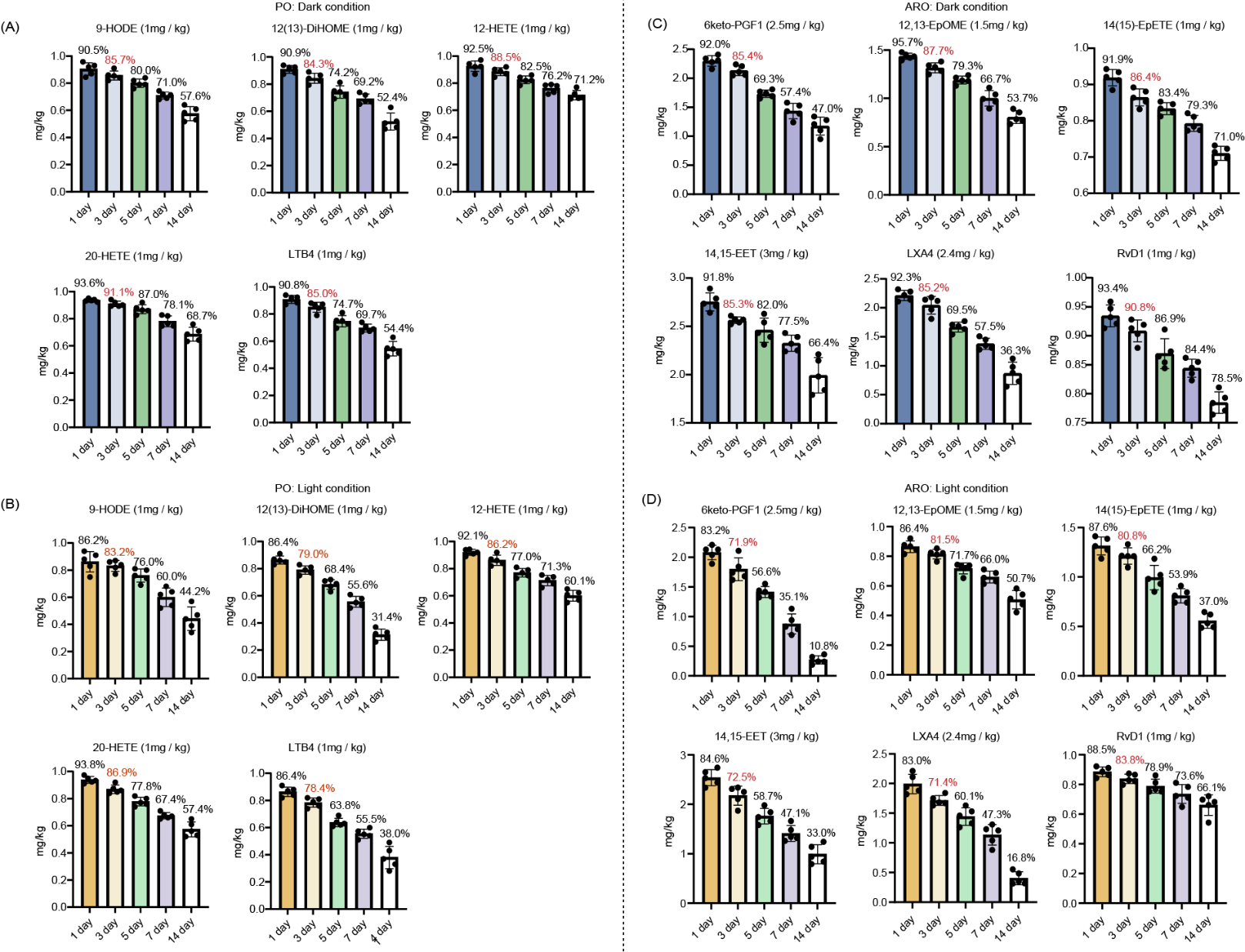


# Figure S8 Time-dependent dynamic assessments of the stability of oxylipins in the diets under dark and light conditions. (A, B), The residual proportions of the top-five proinflammatory oxylipins (PO) in chow on different days (1 day, 3 days, 5 days, 7 days, and 2 weeks) under 24 h-dark and 24 h-light conditions (n = 6 feed-pellets in each group). (C, D), The residual proportions of the top-six ARO in chow on different days (1 day, 3 days, 5 days, 7 days, and 2 weeks) under 24 h dark and 24 h light conditions (n = 6 feed-pellets in each group).


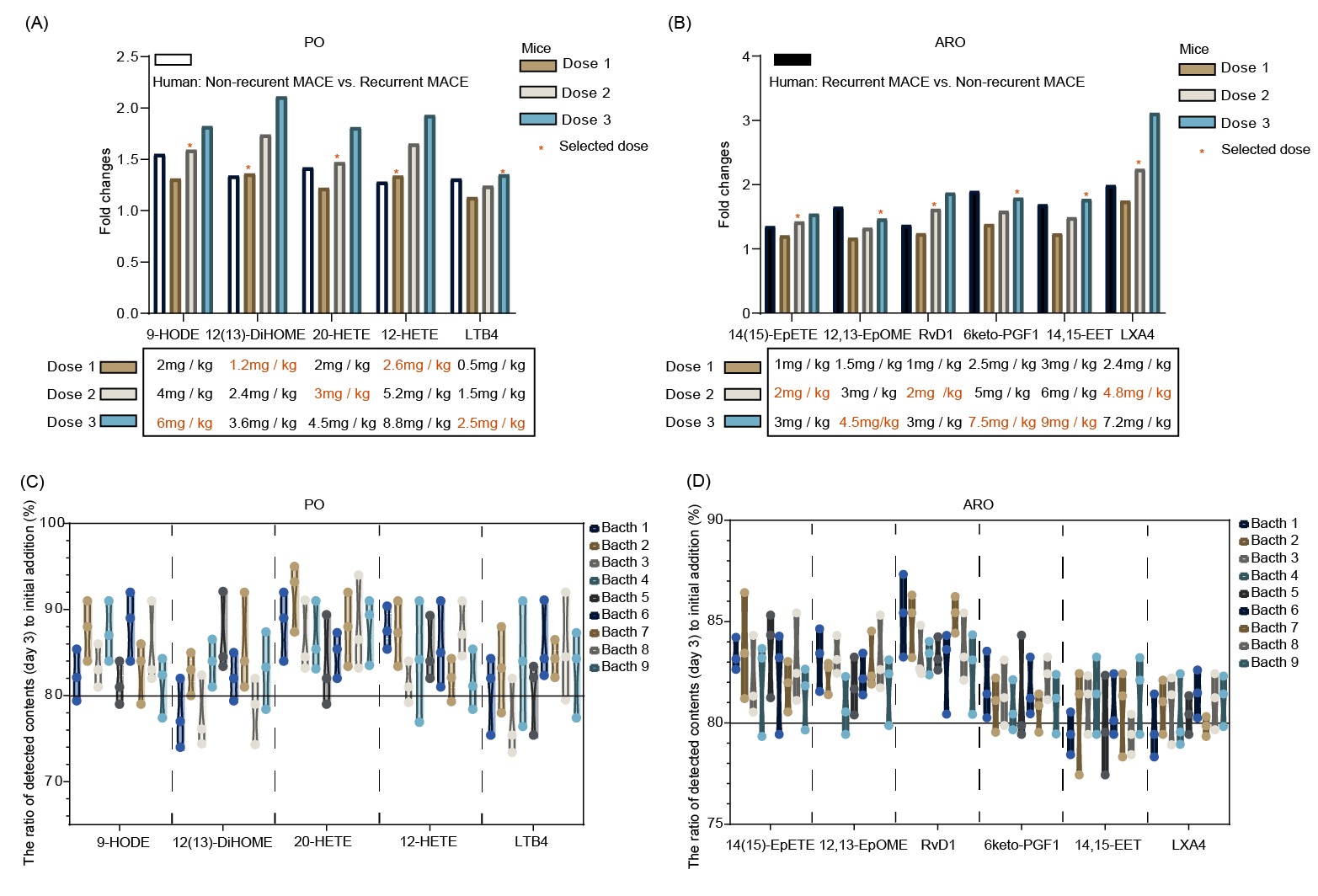


# Figure S9 Optimal dose selection and quality assessment of the oxylipins in the diets. (A, B) Selection of the optimal dose of POs or AROs added to mouse chow. The optimal administered dose was selected on the basis of comparisons of the values of fold change in the plasma levels of mice with and without oxylipin treatment and those of patients with and without recurrent MACE events. (C) The ratio of detected PO levels on day three compared with the initial addition amounts in nine batches of standard mouse chow containing the PO combination (n = 3 feed-pellets in each group). (D) The ratio of the detected ARO levels on day three compared with the initial addition amounts in nine batches of standard mouse chow containing individual ARO (n = 3 feed-pellets in each group).


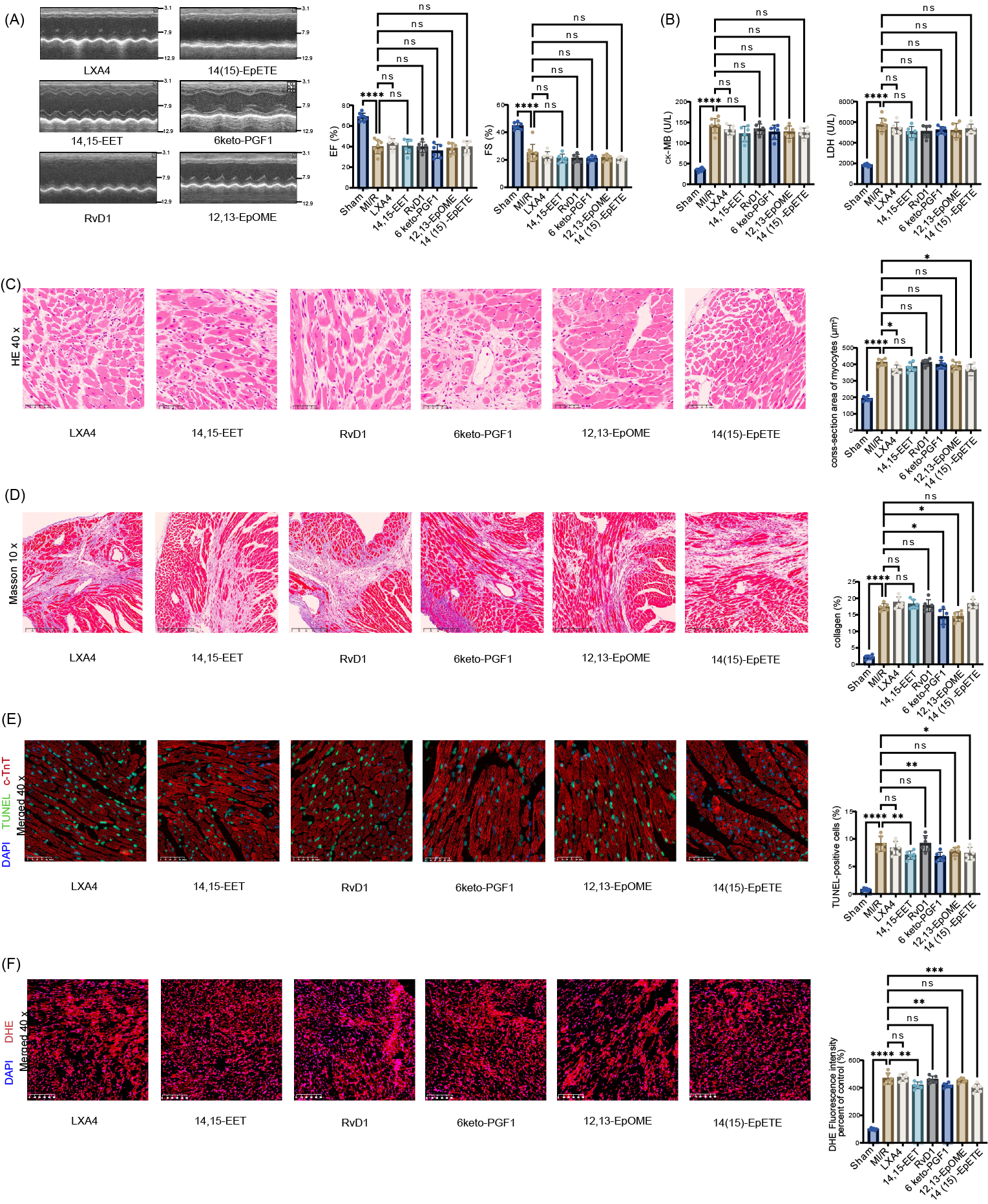


# Figure S10. Evaluation of the effects of six individual ARO treatment on myocardial ischemia‒reperfusion model mice. (A) Representative images of echocardiography and quantitative results (n = 6 mice in each ARO group). (B) Plasma levels of the myocardial injury markers (n = 6 mice in each group), creatine kinase (CK)-MB and lactate dehydrogenase (LDH). (C) Histological analysis of heart tissues stained with hematoxylin-eosin (HE) to demarcate the myocyte surface areas (n = 6 regions in each group). (D) Masson's trichrome for determining the collagen deposition (n = 6 regions in each group). (E) dUTP nick-end labeling (TUNEL)-positive and cardiac troponin (c-TnT) double stained cardiomyocytes (n = 6 regions in each group). (F) Dihydroethidium (DHE)-labeled reactive oxygen species staining of heart tissues (n = 6 regions in each group). **p* < 0.05; ***p* < 0.01, *****p* < 0.0001. Abbreviations: ns represent no statistical differences. EF, ejection fraction; FS, fractional shortening.


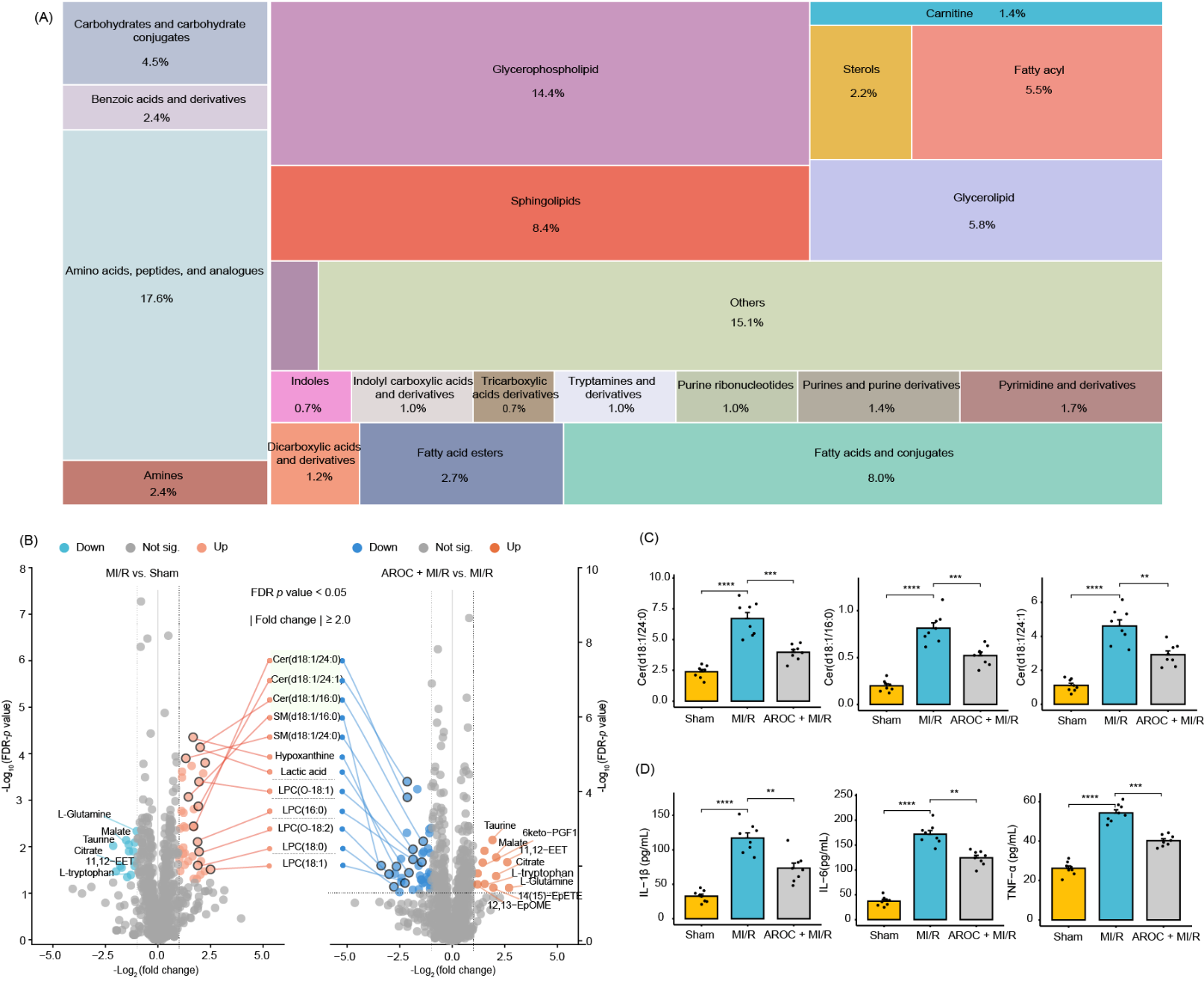


# Figure S11 Effects of the combined treatment of anti-inflammatory/pro-resolving oxylipins on the myocardial metabolome and proinflammatory marker levels in myocardial ischemia‒reperfusion model mice. (A) Treemap overview of the category distribution of all identified metabolites. (B) Volcano plot of the metabolomic alterations in pairwise comparisons of the myocardial ischemia‒reperfusion (MI/R) group vs. the Sham group and the anti-inflammatory/pro-resolving oxylipin combination (AROC) + MI/R group vs. the MI/R group. (C) Plasma concentrations of three ceramides (Cer) after AROC treatment (n = 8 mice in each group). (D) Plasma levels of the pro-inflammatory cytokines after AROC treatment (n = 8 mice in each group). ***p* < 0.01, ****p* < 0.001, *****p* < 0.0001. Abbreviations: IL-1β, interleukin-1β; IL-6, interleukin-6; TNF-α, tumor necrosis factor-alpha; LPC, lysophosphatidylcholine; SM, sphingomyelin; sig., significant.


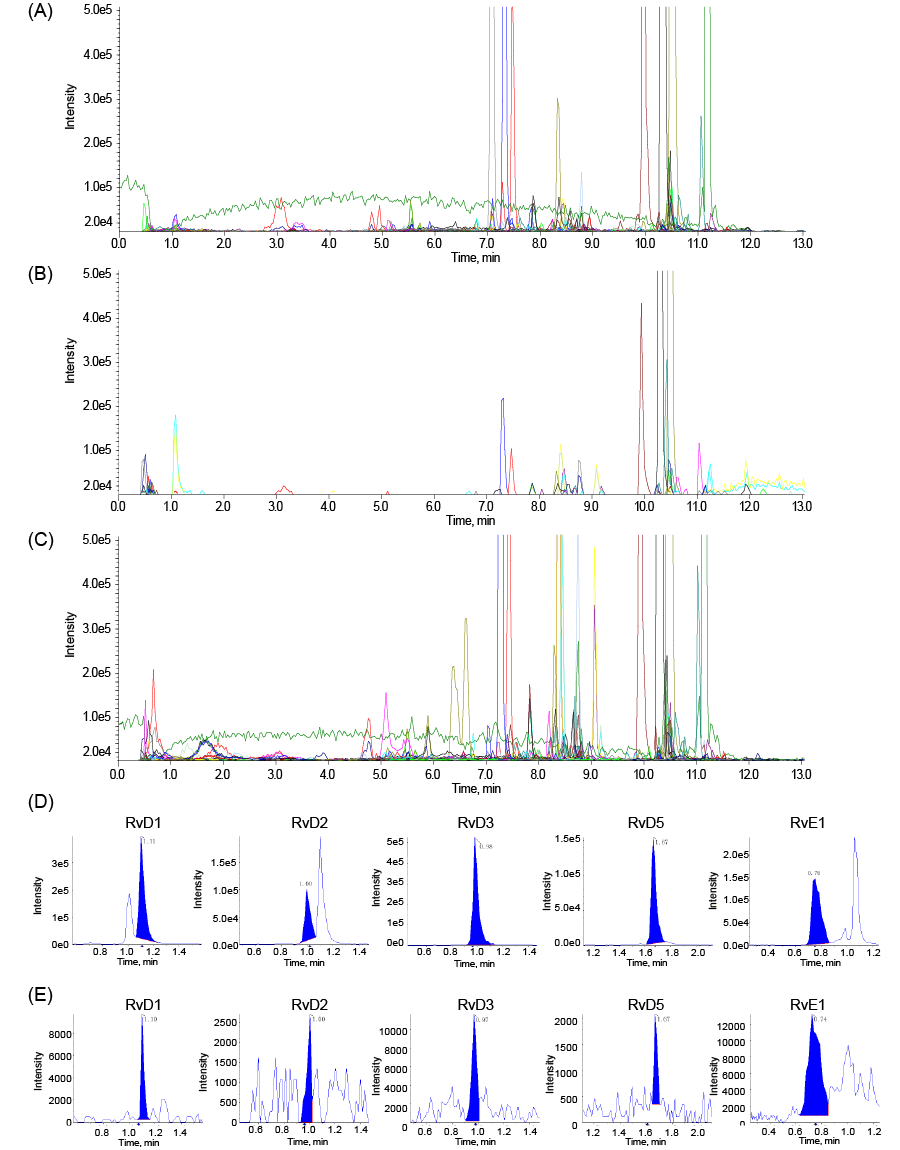


# Figure S12 Optimization of oxylipins extraction methods for plasma sample. (A–C) Representative liquid chromatograph/mass spectrometer chromatograms of solid-phase extraction (A), protein precipitation combined with liquid‒liquid extraction method 1 (B), and protein precipitation combined with liquid‒liquid extraction method 2 (C). (D) Representative chromatograms of the chemical standards of resolvins. (E) The extraction ion chromatogram of resolvins in plasma samples. Abbreviations: RvD1, Resolvin D1; RvD2, resolvin D2; RvD3, resolvin D3; RvD5, resolvin D5; RvE1, resolvin E1.
